# Supplementary material for: Eye health and quality of life: an umbrella review protocol
Source: BMJ Open. 2020 Aug 30;10(8):e037648. doi: 10.1136/bmjopen-2020-037648 (PMC7462163; doi:10.1136/bmjopen-2020-037648)
Supplement: Supplementary data [file bmjopen-2020-037648supp001.pdf]

Online Supplementary File 1

Reporting checklist for protocol of a systematic review
Based on the PRISMA-P guidelines.

		Reporting Item	Page Number
**Title**			
Identification	#1a	Identify the report as a protocol of a systematic review	1
Update	#1b	If the protocol is for an update of a previous systematic review, identify as such	NA
**Registration**			
	#2	If registered, provide the name of the registry (such as PROSPERO) and registration number	6
**Authors**			
Contact	#3a	Provide name, institutional affiliation, e-mail address of all protocol authors; provide physical mailing address of corresponding author	1
Contribution	#3b	Describe contributions of protocol authors and identify the guarantor of the review	11-12
**Amendments**			
	#4	If the protocol represents an amendment of a previously completed or published protocol, identify as such and list changes; otherwise, state plan for documenting important protocol amendments	6
**Support**			
Sources	#5a	Indicate sources of financial or other support for the review	12
Sponsor	#5b	Provide name for the review funder and / or sponsor	12
Role of sponsor or funder	#5c	Describe roles of funder(s), sponsor(s), and / or institution(s), if any, in developing the protocol	12
**Introduction**			

Rationale	#6	Describe the rationale for the review in the context of what is already known	5
Objectives	#7	Provide an explicit statement of the question(s) the review will address with reference to participants, interventions, comparators, and outcomes (PICO)	5
**Methods**			
Eligibility criteria	#8	Specify the study characteristics (such as PICO, study design, setting, time frame) and report characteristics (such as years considered, language, publication status) to be used as criteria for eligibility for the review	6-7
Information sources	#9	Describe all intended information sources (such as electronic databases, contact with study authors, trial registers or other grey literature sources) with planned dates of coverage	7-8
Search strategy	#10	Present draft of search strategy to be used for at least one electronic database, including planned limits, such that it could be repeated	7
Study records - data management	#11a	Describe the mechanism(s) that will be used to manage records and data throughout the review	8
Study records - selection process	#11b	State the process that will be used for selecting studies (such as two independent reviewers) through each phase of the review (that is, screening, eligibility and inclusion in meta-analysis)	8-9
Study records - data collection process	#11c	Describe planned method of extracting data from reports (such as piloting forms, done independently, in duplicate), any processes for obtaining and confirming data from investigators	9-10
Data items	#12	List and define all variables for which data will be sought (such as PICO items, funding sources), any pre-planned data assumptions and simplifications	9-10
Outcomes and prioritization	#13	List and define all outcomes for which data will be sought, including prioritization of main and additional outcomes, with rationale	9-10

Risk of bias in individual studies	#14	Describe anticipated methods for assessing risk of bias of individual studies, including whether this will be done at the outcome or study level, or both; state how this information will be used in data synthesis	9-11
Data synthesis	#15a	Describe criteria under which study data will be quantitatively synthesised	NA
Data synthesis	#15b	If data are appropriate for quantitative synthesis, describe planned summary measures, methods of handling data and methods of combining data from studies, including any planned exploration of consistency (such as I2, Kendall's τ)	NA
Data synthesis	#15c	Describe any proposed additional analyses (such as sensitivity or subgroup analyses, meta-regression)	NA
Data synthesis	#15d	If quantitative synthesis is not appropriate, describe the type of summary planned	10-11
Meta-bias(es)	#16	Specify any planned assessment of meta-bias(es) (such as publication bias across studies, selective reporting within studies)	NA
Confidence in cumulative evidence	#17	Describe how the strength of the body of evidence will be assessed (such as GRADE)	11

The PRISMA-P checklist is distributed under the terms of the Creative Commons Attribution License CC-BY 4.0. This checklist was completed on 06. February 2020 using https://www.goodreports.org/, a tool made by the EQUATOR Network in collaboration with Penelope.ai

Online Supplementary File 2

Search strategy for MEDLINE Ovid

1. exp Eye Diseases/

2. exp Eye Injuries/

3. exp Administration, Ophthalmic/

4. exp Diagnostic Techniques, Ophthalmological/

5. exp Eye Protective Devices/

6. exp Glaucoma Drainage Implants/

7. exp Injections, Intraocular/

8. exp Ophthalmic Solutions/

9. exp Ophthalmologic Surgical Procedures/

10. exp Optical Devices/

11. exp Orbital Implants/

12. exp Orthoptics/

13. exp Pseudophakia/

14. exp Visual Prosthesis/

15. ((low* or handicap* or subnormal* or impair* or partial* or disab* or reduce* or diminish* or decrease*) adj3 (vision or visual* or sight*)).tw.

16. ((abnormal* or blurred or defect* or difficult* or dim or disturbed or hazy or interference or poor or tunnel or weak* or defect* or deficienc* or disorder* or disturb* or problem*) adj3 (vision or visual* or sight*)).tw.

17. ((delayed or agnosia or constriction* or prosthesis or prostheses) adj3 (vision or visual* or sight*)).tw.

18. ((vision or visual or sight*) adj2 loss).tw.

19. (Ocular or occular or intraocular or ophthalmol* or ophthalmic* or ophthalmop* or optic* or orbital or conjunctival or conjunctivitis or eye or eyes or eyelid* or cataract* or corneal or glaucoma* or lacrimal or lacrimation or macular or retinal or retinitis or retinoblastoma or retinopath* or retrobulbar neuritis or uveal or uveitis or vitrectomy or vitreous detachment* or vitreous haemorrhage* or vitreous hemorrhage* or vitreous membranes or vitreous strands or vitreous prolapse* or vitreous syneresis).tw.

20. (Amblyopia or Ametropia or Anisocoria or Anophthalmia or Anterior Chamber Haemorrhage or Anterior Chamber Hemorrhage or Aphakia or Aqueous Outflow Obstruction or Asthenopia or Balint's Syndrome or Blepharitis or Blepharospasm or chalazia or chalazion or Chorioretinal Disorder* or Chorioretinitis or Choroid Diseases or Choroidal or Choroiditis or Chromatopsia or Diplopia or Endophthalmitis or Epiphora or Episcleritis or Equatorial Staphyloma or Esotropia or Exophthalmos or Fixed Pupil* or Fuchs endothelial dystrophy or Hemianopia or Hemianopsia or Hepatolenticular Degeneration or Hordeola or Hordeolum or Horner's Syndrome or Hypopyon or Iritis or Keratitis or Keratoconjunctivitis or Keratoconus or Lens Disease* or Lens Disorder* or Lens Opacit* or Lens Subluxation or Localized Anterior Staphyloma or Meibomianitis or Miosis or Mydriasis or Myopia or Nystagmus or Oculopath* or Papilloedema or Periorbital Fat Herniation or (Periocular and carcinoma*) or Photalgia or Photophobia or Photopsia or Pigment Precipitation or Posterior capsule opacification or Posterior Dislocation Of Lens or Posterior Synechiae or Pseudophakia or Proliferative Vitreoretinopathy or Scleral Disease* or Scleral Staphyloma or Scleritis or Scotoma or Staphyloma Posticum or Strabismus or Symblepharon or Traumatic Hyphema or Wavefront Aberration* or Wegener's granulomatosis or Wilson's Disease or Xerophthalmia or refractive error* or near-sighted* or nearsighted* or short-sighted* or shortsighted* or hyperopia or farsighted* or far sighted* or long-sighted* or longsighted*or astigmatism or presbyopia* or onchocerciasis or onchocerciases).tw.

21. (LASIK or LASEK or Orthoptic* or "visual prosthesis" or "visual prostheses" or "artificial iris" or "capsular tension ring" or "cornea implant" or "intravitreal implant" or "lens implant" or "palpebral spring" or "punctal plug" or "retinal implant" or "sclerectomy implant" or glasses or spectacle* or "artificial lens" or "artificial implant lens" or pseudophakos or "orbit implant" or "ab interno gel implant" or "ab interno gel stent" or "anterior chamber drainage tube" or "aqueous drainage device" or "aqueous drainage implant" or "aqueous shunt" or glaukos or istent or keratoprosthesis).tw.

22. or/1-21

23. "Quality of Life"/ or Quality-Adjusted Life Years/ or "Value of Life"/ or Health Status/ or Sickness Impact Profile/ or Disability Evaluation/ or exp "Activities of Daily Living"/ or Cost-Benefit Analysis/ or "Surveys and Questionnaires"/ or Health surveys/ or exp psychometrics/

24. (quality adj2 life).tw.

25. ("disability adjusted life" or qaly* or qald* or qale* or qtime* or daly* or euroqol or "euro qol" or eq5d or "eq 5d" or hql or hqol or "h qol" or hrqol or "hr qol" or hye or hyes or health* year* equivalent* or hui or hui1 or hui2 or hui3 or "willingness to pay" or "standard gamble" or QOL or HRQL or HRQOL or wellbeing or "well being" or WHOQOL or "healthy days measures" or "EQ VAS" or "EQ 15D" or "36 Item Short Form Survey" or "SF 36" or "12 item Short Form Survey" or "SF 12" or "Visual Function Questionnaire" or "NEI VFQ" or "VFQ 25" or "IND VFQ 33" or "14 item Visual Functioning" or "VF 14" or "11 item Visual Functioning" or "VF 11" or "Impact of Vision Impairment" or IVI or "glaucoma utility index" or catquest or "Activities of Daily Vision Scale" or ADVS or "Cataract Symptom Scale" or "Daily Living Tasks Dependent Upon Vision" or DLTV or "Measure of Outcome in Ocular Disease" or "Refractive Status and Vision Profile" or "Vision Specific Sickness Impact Profile" or SIPV or "Visual Activities Questionnaire*" or VAQ or "Visual

Disability Assessment*" or VDA or "Visual Disabilities Questionnaire*" or "Visual Function Questionnaire*" OR "VA LV VFQ" or "Glaucoma symptom scale" or "Symptom Impact Glaucoma Score" or GHPI or "Glaucoma Health Perceptions index").tw.

26. (health adj3 (utility* or disutili* or state or status)).tw.

27. ((visual or vision) adj3 (disabilit* or disabled or function* or activit* or task or performance or impairment or Questionnaire*)).tw.

28. or/23-27

29. 22 and 28

30. Cochrane Database Syst Rev.ja. or Meta-Analysis.pt. or (Search* or Medline or (Systematic and Review)).tw.

31. limit 29 to systematic reviews

32. 30 or 31

33. exp Animals/ not exp Humans/

34. 32 not 33

35. 29 and 34

BMJ Publishing Group Limited (BMJ) disclaims all liability and responsibility arising from any reliance placed on this supplemental material which has been supplied by the author(s)

Online Supplementary File 3

Joanna Briggs Institute Data Extraction Form for Review for Systematic Reviews and Research Syntheses

Study Details	
Author/Year	
Objectives	
Participants	
Characteristics	
Total number	
Setting/context	
Description of Interventions/phenomena of interest	
Search Details	
Sources searched	
Range (years) of included studies	
Number of studies included	
Types of studies included	
Country of origin of included studies	
Appraisal	
Appraisal instruments used	
Appraisal rating	
Analysis	
Method of analysis	
Outcome assessed	
Results/Findings	
Significance/direction	
Heterogeneity	
Comments	
