# Supplementary material for: Eye health and quality of life: an umbrella review protocol
Source: BMJ Open. 2020 Aug 30;10(8):e037648. doi: 10.1136/bmjopen-2020-037648 (PMC7462163; doi:10.1136/bmjopen-2020-037648)
Supplement: Author's manuscript [file bmjopen-2020-037648.draft_revisions.pdf]

BMJ Open is committed to open peer review. As part of this commitment we make the peer review history of every article we publish publicly available.

When an article is published we post the peer reviewers' comments and the authors' responses online. We also post the versions of the paper that were used during peer review. These are the versions that the peer review comments apply to.

The versions of the paper that follow are the versions that were submitted during the peer review process. They are not the versions of record or the final published versions. They should not be cited or distributed as the published version of this manuscript.

BMJ Open is an open access journal and the full, final, typeset and author-corrected version of record of the manuscript is available on our site with no access controls, subscription charges or pay-per-view fees (http://bmjopen.bmj.com).

If you have any questions on BMJ Open's open peer review process please email
info.bmjopen@bmj.com

BMJ Open

Eye Health and Quality of Life: An Umbrella Review Protocol

Journal:	*BMJ Open*
Manuscript ID	bmjopen-2020-037648
Article Type:	Protocol
Date Submitted by the Author:	10-Feb-2020
Complete List of Authors:	Assi, Lama; Johns Hopkins University School of Medicine, Wilmer Eye Institute<br>Rosman, Lori ; Johns Hopkins University School of Medicine, Welch Medical Library<br>Chamseddine, Fatimah; American University of Beirut Faculty of Medicine, Clinical Research Institute<br>Ibrahim, Perla; American University of Beirut Faculty of Medicine, Department of Ophthalmology<br>Sabbagh, Hadi; American University of Beirut Faculty of Medicine, Department of Ophthalmology<br>Congdon, Nathan; Queen's University Belfast School of Medicine Dentistry and Biomedical Sciences, Centre for Public Health; Sun Yat-Sen University, Zhongshan Ophthalmic Center<br>Evans, Jennifer; London School of Hygiene & Tropical Medicine, International Centre for Eye Health<br>Ramke, Jacqueline; London School of Hygiene & Tropical Medicine, International Centre for Eye Health; The University of Auckland, School of Optometry and Vision Science<br>Kuper, Hannah; London School of Hygiene & Tropical Medicine, International Centre for Evidence in Disability<br>Burton, Matthew J; London School of Hygiene & Tropical Medicine, International Centre for Eye Health; Moorfields Eye Hospital<br>Ehrlich, Joshua; University of Michigan, Department of Ophthalmology and Visual Sciences; University of Michigan, Institute for Healthcare Policy and Innovation<br>Swenor, B; Johns Hopkins University School of Medicine, Wilmer Eye Institute; Johns Hopkins University Bloomberg School of Public Health, Department of Epidemiology
Keywords:	OPHTHALMOLOGY, PUBLIC HEALTH, EPIDEMIOLOGY

SCHOLARONE™
Manuscripts

I, the Submitting Author has the right to grant and does grant on behalf of all authors of the Work (as defined in the below author licence), an exclusive licence and/or a non-exclusive licence for contributions from authors who are: i) UK Crown employees; ii) where BMJ has agreed a CC-BY licence shall apply, and/or iii) in accordance with the terms applicable for US Federal Government officers or employees acting as part of their official duties; on a worldwide, perpetual, irrevocable, royalty-free basis to BMJ Publishing Group Ltd ("BMJ") its licensees and where the relevant Journal is co-owned by BMJ to the co-owners of the Journal, to publish the Work in this journal and any other BMJ products and to exploit all rights, as set out in our licence.

The Submitting Author accepts and understands that any supply made under these terms is made by BMJ to the Submitting Author unless you are acting as an employee on behalf of your employer or a postgraduate student of an affiliated institution which is paying any applicable article publishing charge ("APC") for Open Access articles. Where the Submitting Author wishes to make the Work available on an Open Access basis (and intends to pay the relevant APC), the terms of reuse of such Open Access shall be governed by a Creative Commons licence – details of these licences and which Creative Commons licence will apply to this Work are set out in our licence referred to above.

Other than as permitted in any relevant BMJ Author's Self Archiving Policies, I confirm this Work has not been accepted for publication elsewhere, is not being considered for publication elsewhere and does not duplicate material already published. I confirm all authors consent to publication of this Work and authorise the granting of this licence.

For peer review only

Eye Health and Quality of Life: An Umbrella Review Protocol

Lama Assi[1], MD, Lori Rosman[2], MLS, Fatimah Chamseddine[3], MD, Perla Ibrahim[4], MD, Hadi Sabbagh[4], BS, Nathan Congdon[5,6,7], MD, MPH, Jennifer Evans[8], MSc, PhD, Jacqueline Ramke[8,9], PhD, MPH, Hannah Kuper[10], ScD, Matthew J Burton[8,11], PhD, FRCOphth, Joshua R Ehrlich[12,13], MD, MPH, Bonnielin Swenor[1,14]*, PhD, MPH

Author Affiliations

1. Wilmer Eye Institute, Johns Hopkins University School of Medicine, Baltimore, USA
2. Welch Medical Library, Johns Hopkins University School of Medicine, Baltimore, USA
3. Clinical Research Institute, American University of Beirut Faculty of Medicine, Beirut, Lebanon
4. Department of Ophthalmology, American University of Beirut Faculty of Medicine, Beirut, Lebanon
5. Centre for Public Health, Queen's University Belfast School of Medicine Dentistry and Biomedical Sciences, Belfast, UK
6. Orbis International, New York, United States
7. Zhongshan Ophthalmic Center, Sun Yat-sen University, Guangzhou, China
8. International Centre for Eye Health, London School of Hygiene & Tropical Medicine, London, UK
9. School of Optometry and Vision Science, University of Auckland, Auckland, New Zealand
10. International Centre for Evidence in Disability, London School of Hygiene & Tropical Medicine, London, UK
11. Moorfields Eye Hospital, London, UK
12. Department of Ophthalmology and Visual Sciences, University of Michigan, Ann Arbor, MI, USA
13. Institute for Healthcare Policy and Innovation, University of Michigan, Ann Arbor, MI, USA
14. Department of Epidemiology, Johns Hopkins Bloomberg School of Public Health, Baltimore, MD, USA

*Corresponding author:
Bonnielin Swenor
bswenor@jhmi.edu
600 N. Wolfe Street
Wilmer 116
Baltimore, MD 21287
Phone: 410-614-2874

Word count: 2596

ABSTRACT

Introduction: Vision impairment and eye disease are major global health concerns and have been associated with increased morbidity and mortality, and lower quality of life. Quality of life, whether generic, vision-specific, or disease-specific, is an important measure of the impact of eye health on people's daily activities, well-being, and visual function, and is increasingly used to evaluate the impact of ophthalmic interventions and new devices. While many studies and reviews have examined the relationship between vision or eye health and quality of life across different contexts, there has yet to be a synthesis of the impact of vision impairment, eye disease, and ophthalmic interventions on quality of life globally and across the lifespan.

Methods and analysis: An umbrella review of systematic reviews will be conducted to address these two questions: 1) What is the association of vision impairment and eye disease with quality of life; and 2) What is the impact of ophthalmic interventions on quality of life? A search of related literature will be performed in Medline Ovid, Embase.com, Cochrane Database of Systematic Reviews, Proquest Dissertations and Theses Global, and the grey literature. Title and abstract screening, full-text eligibility and methodological quality assessment, and data extraction will be conducted by reviewers working independently and in duplicate. Assessment of methodological quality and data extraction will be performed using Joanna Briggs Institute standard forms. Findings from the systematic reviews and their methodological quality will be summarized qualitatively in the text and using tables.

Ethics and dissemination: No ethical approval is required. Results of this umbrella review will be published in a peer-reviewed journal and summarized in the *Lancet Global Health* Commission on Global Eye Health.

Registration: This protocol was registered in the Open Science Framework Registries (https://osf.io/qhv9g/) on the 10th February 2020.

Keywords: ophthalmology, public health, epidemiology

For peer review only

ARTICLE SUMMARY

Strengths and limitations of this study:

- The umbrella review approach allows for a comprehensive review of a very broad topic by summarizing the evidence from multiple research syntheses into one systematic review of reviews.
- Study screening, critical appraisal, and data collection will be conducted in duplicate.
- Standardized forms developed specifically for the conduct of umbrella reviews will be used for critical appraisal and data collection.
- Studies related to rare topics or special settings might not be included in systematic reviews, and thus would not be represented in this umbrella review, a main limitation of our work.
- Only systematic reviews published in English will be included.

For peer review only

INTRODUCTION

Vision impairment is a major cause of disability worldwide.[1] In 2015, an estimated 36 million people were blind, 217 million had moderate or severe vision impairment, and over a billion people experienced near-vision impairment (presbyopia).[2] Cataract and uncorrected refractive error are correctable conditions which accounted for 78% of global visual impairment that year.[3] Despite reductions in age-specific prevalence, the number of people with vision impairment and blindness is projected to increase due to population growth and aging.[2] Vision impairment is associated with negative health outcomes, such as having multiple chronic conditions,[4,5] and increased mortality,[6] and also induces substantial socioeconomic consequences for individuals,[7] and an associated lower quality of life.

Objective clinical measures, like visual acuity, intraocular pressure, or fundus imaging, are widely used in the clinical and research settings to assess eye health, but often fail to capture the impact of vision impairment or eye disease on individuals' daily activities or social well-being.[8] Quality of life instruments, on the other hand, measure patient-reported outcomes, such as perceived health, physical, mental, emotional, or social well-being, and even vision-specific function. These measures are important as vision impairment can have a large impact on quality of life, possibly to an even greater degree than major conditions such as stroke, heart disease, or diabetes.[9] Both severe conditions that lead to marked reduction in vision like age-related macular degeneration,[10] and highly symptomatic conditions which may not be associated with impaired vision, like dry eye syndrome,[11] have been associated with decreased health-related quality of life.

The use of quality of life instruments has gained popularity in ophthalmic studies, including clinical trials, over the past decade.[12] While there is a wide range of quality of life instruments available, vision-related quality of life instruments are frequently used in ophthalmic studies, as these questionnaires are more sensitive to the impact of subtle vision changes on daily function compared to more general health-related or generic quality of life tools.[13] Reduced visual acuity[14] and visual field loss[15] are both associated with worsening in vision-related quality of life; and glaucoma[16-19] and cataract[16,19] are associated with worse vision-related function, independent of visual acuity. In ophthalmic clinical trials, health-related, vision-related, and even disease-specific scales have been used as secondary outcome measures, and more recently, as primary outcomes as well.[20,21] Patient-reported outcomes are also increasingly being incorporated in the evaluation of new ophthalmic devices, and the

For peer review only

Food and Drug Administration even provides guidance on using them to support labeling claims too.[22,23]

There has yet to be a global assessment of the impact of eye health, including vision impairment, eye disease, and ophthalmic interventions on quality of life across the lifespan, despite the growing number of ophthalmic studies assessing quality of life, and increased value placed on patient-reported outcomes. Prior studies on vision and quality of life have usually focused on specific countries (e.g., USA,[24] Finland,[25] South Korea,[26] Nigeria[27]), populations (e.g., Malay population in Singapore,[28] Latino population[29] and indigenous peoples of the Americas[30] in the USA), or settings (e.g., community,[31] outpatient clinics[32]). Even reviews summarizing the evidence about the impact of vision on quality of life have usually focused on specific age groups (e.g., children,[33,34] older adults[35]), eye conditions (e.g., glaucoma,[36] diabetic retinopathy,[37] dry eye[38]), or interventions (e.g., low vision rehabilitation for children,[34] anti-VEGF treatment for age-related macular degeneration[39]).

This umbrella review (or systematic review of systematic reviews) will examine the impact of vision impairment, eye disease and ophthalmic interventions on quality of life globally and across the lifespan. An umbrella review approach allows us to maintain a broad scope while relying on the highest quality of evidence, given the large number of primary studies[9-11,14-19,24-32] and reviews on this topic[34-41]. A search of three systematic review registries (PROSPERO, Joanna Briggs Institute Systematic Review Register, and Open Science Framework Registries) has shown that there is currently no systematic or umbrella review underway for this topic.

Objectives and Questions

This umbrella review of systematic reviews aims to identify and synthesize currently available knowledge about the association of vision and eye disease with quality of life on a global level. Two questions will be addressed:

1) What is the association between vision impairment or eye disease and quality of life?
2) What is the impact of ophthalmic interventions on quality of life?

For peer review only

METHODS AND ANALYSIS

This protocol was registered in the Open Science Framework Registries (https://osf.io/qhv9g/) on the 10th February 2020. It was designed by following the Joanna Briggs Institute guidelines for the conduct and preparation of umbrella reviews,[42] and the Preferred Reporting Items for Systematic review and Meta-Analysis Protocols (PRISMA-P) guidelines for the reporting of systematic review protocols (online supplementary file 1).[43] The anticipated start date of this study is the 11th February 2020. Any changes to the methodological approach will be dated and described in detail in the final umbrella review report.

Inclusion Criteria

Systematic reviews and meta-analyses that evaluate the impact of vision impairment, eye disease, or ophthalmic interventions on quality of life will be included in this umbrella review.

Types of participants

Systematic reviews of studies with participants who have vision impairment, or an eye disease will be included. Vision impairment can be self-reported or assessed objectively, using any measure of visual function, including, but not limited to, visual acuity (corrected or uncorrected, distance or near), contrast sensitivity, or visual fields. Eye disease diagnosis can be based on self-report, medical chart or claims data, or an objective assessment of symptoms, clinical signs, or imaging findings. Eye diseases that will be explored include, but are not limited to, the World Health Organization (WHO) priority eye diseases, which are the most common causes of vision impairment worldwide: cataract, onchocerciasis, trachoma, refractive errors, age-related macular degeneration, diabetic retinopathy, glaucoma, corneal opacities, childhood blindness, and genetic eye diseases.[44]

Systematic reviews with sample populations from any age group (children, working-age adults, or older adults), country (low, middle or high income), or setting (community, hospital, clinic, institution) will be included.

For peer review only

Interventions

Systematic reviews that examine interventions will also be included and will help answer the second question specifically, the impact of ophthalmic interventions on quality of life. The comparison group can be the same group pre-intervention, another group that receives another intervention, or another group receiving no intervention. Any ophthalmic intervention identified will be included, as long as its main aim is to correct or improve vision, slow down the progression of vision loss, improve functional ability among those with vision loss (e.g., low vision rehabilitation, use of assistive devices), or relieve eye pain or discomfort.

Outcomes

Studies that measure any aspect of quality of life (generic or health-related, vision-related, or disease-specific) will be included. Studies can report on one or all domains used to measure quality of life. Systematic reviews of both quantitative and qualitative studies are eligible for inclusion. Examples of quality of life instruments are the WHO Quality of Life Assessment Instrument (health-related quality of life), the National Eye Institute Vision Function Questionnaire (vision-related quality of life), and the Catquest-9SF questionnaire (cataract-specific quality of life).

Types of studies

Only systematic reviews (with or without and meta-analyses) are eligible for inclusion. A systematic review will be defined as a review that includes every one of these items: a research question, a search strategy with the sources searched, inclusion and exclusion criteria, screening methods, a discussion about the quality of included studies and risk of bias, and information about data analysis and synthesis.[45] Systematic reviews of observational and interventional studies will be included, but those that incorporate case series or expert opinion as their source of evidence will be excluded. All other types of reviews, including narrative reviews and scoping reviews will be excluded.

Search Strategy

An academic librarian developed a comprehensive search strategy based on similar ones used by Cochrane Eyes and Vision Group. Search strategies were developed using a combination of controlled vocabulary and keywords to represent vision terms, eye diseases, including all

For peer review only

the WHO priority eye disease listed above, and ophthalmic interventions, as well as search terms for quality of life, including some commonly used scales, and terms to identify systematic reviews (see Online Supplementary File 2 for a detailed search strategy).

The following databases will be searched: Medline Ovid (1946 to present), Embase.com (1947 to present), Cochrane Database of Systematic Reviews (1995 to present), Proquest Dissertations and Theses Global (1861 to present). A search for grey literature will include sources such as reports from governments and non-governmental organizations, and databases including the Open Grey and the Agency for Healthcare Research and Quality. The search will be limited to articles published in English with no restrictions on the year of publication. We will search the references of included studies for additional systematic reviews.

The search will be run again in the synthesis stage to identify any relevant reviews published since the initial search.

Study Selection

Citations retrieved from the searches will be imported to Endnote, where any duplicates will be removed. Then, references will be imported to Covidence, a web-based software platform that streamlines the production of systematic reviews. Four reviewers will work in pairs to screen the studies independently and in duplicate. Conflicts will be discussed in the presence of a third reviewer from the second pair; if no consensus is reached, the senior author will be consulted.

Review selection will take place at the level of title/abstract and full text. Reviews judged to be of insufficient quality for inclusion will also be excluded at the methodological assessment stage. Articles will first be screened at the level of the titles and abstracts. At this stage, all systematic reviews and meta-analyses, published in English, and that address a vision-related topic (vision impairment, eye disease, or ophthalmic intervention) and quality of life will be included. Articles that are identified as systematic reviews in the title or abstract, using the terms “systematic review” or “meta-analysis”, will be included. Reviews that are not explicitly identified as such will be moved to full-text review if the methods suggest they may be systematic reviews based on the definition used above. Reviews that are clearly not

For peer review only

related to quality of life will be excluded at this stage, but reviews of interventional studies that do not specifically address quality of life in their title or abstract will go to full-text review, as quality of life may be a secondary outcome that is only mentioned in the text.

In the next step, the included reviews will undergo full-text screening and will be included if they meet the criteria listed above for a systematic review, if none of their primary studies are case series/case reports or expert opinion, and if they specifically assess the impact of the vision-related topic on quality of life. The final study selection will be made after assessment of methodological quality. Reasons for exclusion will be logged.

Assessment of Methodological Quality

Systematic reviews that are deemed eligible for inclusion will be assessed for their methodological quality using the JBI Critical Appraisal Checklist for Systematic Reviews and Research Syntheses.[46] The four reviewers will work again in pairs to do the assessment independently and in duplicate. JBI SUMARI, a web-based review software that has partnered with Covidence, will be used to facilitate the critical appraisal step.

The JBI Critical Appraisal Checklist contains 11 items related to systematic review methodology, each graded as “Yes”, “No”, “Unclear”, or “Not Applicable”. It can be used to appraise both quantitative and qualitative systematic reviews. The form will be piloted by the four reviewers by testing it on two studies before starting independent appraisals; these reviewers will compare their results and discuss what constitutes an acceptable level of information to decide if a review meets or does not meet the criteria, and when is it “Unclear”. Systematic reviews for which at least one of the items “clear review question”, “appropriate inclusion criteria”, “appropriate search strategy”, or “appropriate criteria for critical appraisal” are graded as “Unclear” or “No” will be considered to be of insufficient quality for inclusion, and as such would be excluded at this stage.

Results of the quality appraisal for each review will be presented in a table, and the overall methodological quality of the included reviews will be summarized in the text.

For peer review only

Data Collection

Data will be extracted from the final list of articles included using the JBI Data Extraction Form for Review for Systematic Reviews and Research Syntheses.[42] Again, the four reviewers will work in pairs to extract the data independently and in duplicate, using the JBI SUMARI software.

In brief, the standardized form will be used to extract information about citation details (e.g., author, year), systematic review methodology (e.g., objectives, participants, setting/context, search strategy, appraisal instrument used), characteristics of the included studies (e.g., date range, number and types of studies, country of origin, rating of their quality, outcomes reported), and findings of the systematic review (e.g., method of synthesis/analysis employed, results/findings). Additionally, information about the review's funder or sponsor and their role, when applicable, and the reviewers' overall assessment of the quality of the evidence, such as GRADE (Grading of Recommendations Assessment, Development, and Evaluation), will be collected. The GRADE quality assessment is based on the primary studies' quality and design, and the consistency and directness of the findings.[47]

In the comments section, the following information will be indicated: 1) if the review is about vision impairment/eye health or an ophthalmic intervention, 2) the functional vision measure used or eye disease or intervention, 3) if the population belongs to a specific age group, country income group, or setting. This will help classify the reviews for the synthesis.

Before the reviewers start collecting data independently, the form will be piloted. All four reviewers will extract data from two articles, compare their answers and discuss them to ensure that they all interpret the questions in the same way.

Data Summary

A qualitative synthesis of the findings will be presented in the text and using tables describing study characteristics and the overall umbrella review results (summary of findings). When presenting study characteristics, studies will first be divided according to the question they address: the impact of vision impairment or eye disease on quality of life, or the impact of ophthalmic interventions on quality of life. Each question will have a table for the quantitative systematic reviews, and another one for the qualitative systematic reviews, as the

For peer review only

study characteristic information reported/presented for each are different. Within each table, results will be stratified according to the outcome measured (health-related, vision-related, or condition-specific quality of life), and functional vision measured, eye disease, or intervention (Figure 1).

Study characteristics tables for the quantitative systematic reviews will include the following information: the number of studies in the systematic review, the number of participants from the included studies, estimates computed, and the heterogeneity of the results. For qualitative systematic reviews, the final synthesized findings will be presented along with information about the study context. Overlaps of original research studies in the included systematic reviews will be presented.

Summary of findings tables will present an overall summary for each question, exposure, outcome, and, when applicable, subgroup (age, country income group, setting) (Figure 1), along with an assessment of the strength of the evidence for each finding, such as GRADE, when included in the review.

Patient and Public Involvement

There is no patient or public involvement in this study.

ETHICS AND DISSEMINATION

Only published studies will be examined for this systematic review; therefore, no ethical approval is required. If any changes to the protocol are made, they will be described in the final umbrella review report.

Results from this study will be published in a peer-reviewed journal and summarized in *The Lancet Global Health* Commission on Global Eye Health.

Figure Legends

Figure 1: Organization of Findings. Reviews will be divided according to the question they address (question 1 being vision impairment/eye disease, and question 2, ophthalmic interventions). For each question, reviews will be categorized as quantitative or qualitative, and within each category, they will be further grouped based on their quality of life measure and exposure (specific functional vision measure or eye disease for question 1, and

For peer review only

intervention for question 2). Summary of findings tables will be further stratified by study population depending on the results available; potential subgroups include age category (children, working-age adults, or older adults), country (low, middle or high income), or setting (community, hospital, clinic, institution).

Acknowledgments: None.

Author Contributions: BS is the guarantor of the review. LA, LR and BS drafted the manuscript. All authors contributed to the development of the selection criteria, the risk of bias assessment strategy and data extraction criteria. LR developed the search strategy. NC, JRE, JR, HK, MJB, and JE provided expertise on systematic and umbrella review methodology, and global eye health. All authors read, provided feedback and approved the final manuscript.

Funding Statement: BS is supported by the National Institutes of Health (NIA K01AG052640). MJB is supported by the Wellcome Trust (207472/Z/17/Z). JR is a Commonwealth Rutherford Fellow, funded by the UK government through the Commonwealth Scholarship Commission in the UK. JRE is supported by the National Institutes of Health (K23EY027848). The Lancet Global Health Commission on Global Eye Health is supported by The Queen Elizabeth Diamond Jubilee Trust, Moorfields Eye Charity [grant number GR001061], NIHR Moorfields Biomedical Research Centre, The Wellcome Trust, Sightsavers, The Fred Hollows Foundation, The SEVA Foundation, The British Council for the Prevention of Blindness and Christian Blind Mission. No funder had any role in the design or conduct of this work.

Competing Interests Statement: All authors declare that they have no conflict of interest.

For peer review only

REFERENCES

1. GBD 2017 Disease and Injury Incidence and Prevalence Collaborators. Global, regional, and national incidence, prevalence, and years lived with disability for 354 diseases and injuries for 195 countries and territories, 1990-2017: A systematic analysis for the global burden of disease study 2017. *Lancet*. 2018;392(10159):1789-1858. doi: S0140-6736(18)32279-7 [pii].

2. Bourne RRA, Flaxman SR, Braithwaite T, et al. Magnitude, temporal trends, and projections of the global prevalence of blindness and distance and near vision impairment: A systematic review and meta-analysis. *Lancet Glob Health*. 2017;5(9):e888-e897. doi: S2214-109X(17)30293-0 [pii].

3. Flaxman SR, Bourne RRA, Resnikoff S, et al. Global causes of blindness and distance vision impairment 1990-2020: A systematic review and meta-analysis. *Lancet Glob Health*. 2017;5(12):e1221-e1234. doi: S2214-109X(17)30393-5 [pii].

4. Garin N, Olaya B, Lara E, et al. Visual impairment and multimorbidity in a representative sample of the spanish population. *BMC Public Health*. 2014;14:815-815. doi: 10.1186/1471-2458-14-815 [doi].

5. Court H, McLean G, Guthrie B, Mercer SW, Smith DJ. Visual impairment is associated with physical and mental comorbidities in older adults: A cross-sectional study. *BMC Med*. 2014;12:181-7. doi: 10.1186/s12916-014-0181-7 [doi].

6. Kuper H, Mathenge W, Macleod D, et al. Mortality during 6 years of follow-up in relation to visual impairment and eye disease: Results from a population-based cohort study of people aged 50 years and above in nakuru, kenya. *BMJ Open*. 2019;9(6):e029700. http://bmjopen.bmj.com/content/9/6/e029700.abstract. doi: 10.1136/bmjopen-2019-029700.

7. Cumberland PM, Rahi JS, UK Biobank Eye and Vision Consortium. Visual function, social position, and health and life chances: The UK biobank study. *JAMA Ophthalmol*. 2016;134(9):959-966. doi: 10.1001/jamaophthalmol.2016.1778 [doi].

8. Denniston AK, Kyte D, Calvert M, Burr JM. An introduction to patient-reported outcome measures in ophthalmic research. *Eye*. 2014;28(6):637-645. https://doi.org/10.1038/eye.2014.41. doi: 10.1038/eye.2014.41.

9. Chia E, Wang JJ, Rochtchina E, Smith W, Cumming RR, Mitchell P. Impact of bilateral visual impairment on health-related quality of life: The blue mountains eye study. *Invest Ophthalmol Vis Sci*. 2004;45(1):71-76. Accessed Jan 21, 2020. doi: 10.1167/iovs.03-0661.

10. Knudtson MD, Klein BEK, Klein R, Cruickshanks KJ, Lee KE. Age-related eye disease, quality of life, and functional activity. *Arch Ophthalmol*. 2005;123(6):807-814. Accessed Jan 21, 2020. doi: 10.1001/archopht.123.6.807.

11. Paulsen AJ, Cruickshanks KJ, Fischer ME, et al. Dry eye in the beaver dam offspring study: Prevalence, risk factors, and health-related quality of life. *Am J Ophthalmol*. 2014;157(4):799-806. Accessed Jan 21, 2020. doi: 10.1016/j.ajo.2013.12.023.

For peer review only

12. Braithwaite T, Calvert M, Gray A, Pesudovs K, Denniston AK. The use of patient-reported outcome research in modern ophthalmology: Impact on clinical trials and routine clinical practice. *Patient Relat Outcome Meas*. 2019;10:9-24. Accessed Jan 21, 2020. doi: 10.2147/PROM.S162802.

13. Margolis MK, Coyne K, Kennedy-Martin T, Baker T, Schein O, Revicki DA. Vision-specific instruments for the assessment of health-related quality of life and visual functioning: A literature review. *Pharmacoeconomics*. 2002;20(12):791-812. Accessed Jan 21, 2020. doi: 10.2165/00019053-200220120-00001.

14. McKean-Cowdin R, Varma R, Hays RD, Wu J, Choudhury F, Azen SP. Longitudinal changes in visual acuity and health-related quality of life: The los angeles latino eye study. *Ophthalmology*. 2010;117(10):1900-1907, 1907.e1. Accessed Jan 21, 2020. doi: 10.1016/j.ophtha.2010.01.059.

15. Patino CM, Varma R, Azen SP, Conti DV, Nichol MB, McKean-Cowdin R. The impact of change in visual field on health-related quality of life the los angeles latino eye study. *Ophthalmology*. 2011;118(7):1310-1317. Accessed Jan 21, 2020. doi: 10.1016/j.ophtha.2010.12.018.

16. Chiang PPC, Zheng Y, Wong TY, Lamoureux EL. Vision impairment and major causes of vision loss impacts on vision-specific functioning independent of socioeconomic factors. *Ophthalmology*. 2013;120(2):415-422. Accessed Jan 21, 2020. doi: 10.1016/j.ophtha.2012.07.077.

17. Lamoureux EL, Chong EW, Thumboo J, et al. Vision impairment, ocular conditions, and vision-specific function: The singapore malay eye study. *Ophthalmology*. 2008;115(11):1973-1981. Accessed Jan 21, 2020. doi: 10.1016/j.ophtha.2008.05.005.

18. Fenwick EK, Ong PG, Man REK, et al. Vision impairment and major eye diseases reduce vision-specific emotional well-being in a chinese population. *Br J Ophthalmol*. 2017;101(5):686-690. Accessed Jan 21, 2020. doi: 10.1136/bjophthalmol-2016-308701.

19. Nirmalan PK, Tielsch JM, Katz J, et al. Relationship between vision impairment and eye disease to vision-specific quality of life and function in rural india: The aravind comprehensive eye survey. *Invest Ophthalmol Vis Sci*. 2005;46(7):2308-2312. Accessed Jan 21, 2020. doi: 10.1167/iovs.04-0830.

20. Lamoureux EL, Mcintosh R, Constantinou M, et al. Comparing the effectiveness of selective laser trabeculoplasty with topical medication as initial treatment (the glaucoma initial treatment study): Study protocol for a randomised controlled trial. *Trials*. 2015;16:406. https://www.ncbi.nlm.nih.gov/pubmed/26362541 https://www.ncbi.nlm.nih.gov/pmc/articles/PMC4567808/. doi: 10.1186/s13063-015-0924-6.

21. Gazzard G, Konstantakopoulou E, Garway-Heath D, et al. Laser in glaucoma and ocular hypertension (LiGHT) trial. A multicentre, randomised controlled trial: Design and methodology. *Br J Ophthalmol*. 2018;102(5):593-598. Accessed Jan 21, 2020. doi: 10.1136/bjophthalmol-2017-310877.

22. NEI and FDA address the use of patient reported outcomes in medical product development. www.eyeresearch.org Web site. www.eyeresearch.org/events/NEI_FDA_PRO_Symposium. Updated 2013. Accessed February 3, 2020.

23. U.S. Food and Drug Administration. Guidance for industry patient-reported outcome measures: Use in medical product development to support labeling claims. www.fda.gov Web site. https://www.fda.gov/regulatory-information/search-fda-guidance-documents/patient-reported-outcome-measures-use-medical-product-development-support-labeling-claims. Updated 2009. Accessed February 3, 2020.

24. Crews JE, Chou CF, Zack MM, et al. The association of health-related quality of life with severity of visual impairment among people aged 40-64 years: Findings from the 2006-2010 behavioral risk factor surveillance system. *Ophthalmic Epidemiol*. 2016;23(3):145-153. doi: 10.3109/09286586.2016.1168851 [doi].

25. Taipale J, Mikhailova A, Ojamo M, et al. Low vision status and declining vision decrease health-related quality of life: Results from a nationwide 11-year follow-up study. *Qual Life Res*. 2019;28(12):3225-3236. doi: 10.1007/s11136-019-02260-3 [doi].

26. Park Y, Shin JA, Yang SW, et al. The relationship between visual impairment and health-related quality of life in korean adults: The korea national health and nutrition examination survey (2008–2012). *PLOS ONE*. 2015;10(7):e0132779. https://doi.org/10.1371/journal.pone.0132779.

27. Bekibele CO, Gureje O. Impact of self-reported visual impairment on quality of life in the ibadan study of ageing. *Br J Ophthalmol*. 2008;92(5):612-615. doi: 10.1136/bjo.2007.124859 [doi].

28. Tong L, Waduthantri S, Wong TY, et al. Impact of symptomatic dry eye on vision-related daily activities: The singapore malay eye study. *Eye (Lond)*. 2010;24(9):1486-1491. doi: 10.1038/eye.2010.67 [doi].

29. Sandhu RK, Munoz BE, Swenor BK, West SK. Refractive error and visual function difficulty in a latino population. *Ophthalmology*. 2012;119(9):1731-1736. doi: 10.1016/j.ophtha.2012.03.003 [doi].

30. McClure TM, Choi D, Becker T, Cioffi GA, Mansberger SL. The effect of visual impairment on vision-related quality of life in american indian/alaska natives. *Ophthalmic Epidemiol*. 2009;16(2):128-135. https://doi.org/10.1080/09286580902745428. doi: 10.1080/09286580902745428.

31. Le Q, Zhou X, Ge L, Wu L, Hong J, Xu J. Impact of dry eye syndrome on vision-related quality of life in a non-clinic-based general population. *BMC Ophthalmol*. 2012;12:22-22. doi: 10.1186/1471-2415-12-22 [doi].

32. Adigun K, Oluleye TS, Ladipo MM, Olowookere SA. Quality of life in patients with visual impairment in ibadan: A clinical study in primary care. *J Multidiscip Healthc*. 2014;7:173-178. doi: 10.2147/JMDH.S51359 [doi].

For peer review only

33. Castañeda YS, Cheng-Patel C, Leske DA, et al. Quality of life and functional vision concerns of children with cataracts and their parents. *Eye*. 2016;30(9):1251-1259. https://www.ncbi.nlm.nih.gov/pubmed/27391939 https://www.ncbi.nlm.nih.gov/pmc/articles/PMC5023803/. doi: 10.1038/eye.2016.134.

34. Elsman EBM, Al Baaj M, van Rens, Gerardus H. M. B., et al. Interventions to improve functioning, participation, and quality of life in children with visual impairment: A systematic review. *Survey of Ophthalmology*. 2019;64(4):512-557. http://www.sciencedirect.com/science/article/pii/S0039625718301516. doi: https://doi.org/10.1016/j.survophthal.2019.01.010.

35. Nyman SR, Dibb B, Victor CR, Gosney MA. Emotional well-being and adjustment to vision loss in later life: A meta-synthesis of qualitative studies. *Disabil Rehabil*. 2012;34(12):971-981. https://doi.org/10.3109/09638288.2011.626487. doi: 10.3109/09638288.2011.626487.

36. Quaranta L, Riva I, Gerardi C, Oddone F, Floriano I, Konstas AGP. Quality of life in glaucoma: A review of the literature. *Adv Ther*. 2016;33(6):959-981. https://doi.org/10.1007/s12325-016-0333-6. doi: 10.1007/s12325-016-0333-6.

37. Khoo K, Man REK, Rees G, Gupta P, Lamoureux EL, Fenwick EK. The relationship between diabetic retinopathy and psychosocial functioning: A systematic review. *Quality of Life Research*. 2019;28(8):2017-2039. https://doi.org/10.1007/s11136-019-02165-1. doi: 10.1007/s11136-019-02165-1.

38. McDonald M, Patel DA, Keith MS, Snedecor SJ. Economic and humanistic burden of dry eye disease in europe, north america, and asia: A Systematic literature review. *The Ocular Surface*. 2016;14(2):144-167. http://www.sciencedirect.com/science/article/pii/S1542012415002049. doi: https://doi.org/10.1016/j.jtos.2015.11.002.

39. Boyle J, Vukicevic M, Koklanis K, Itsiopoulos C. Experiences of patients undergoing anti-VEGF treatment for neovascular age-related macular degeneration: A systematic review. *Psychol Health Med*. 2015;20(3):296-310. Accessed Jan 21, 2020. doi: 10.1080/13548506.2014.936886.

40. Rose MA, Vukicevic M, Koklanis K, Rees G, Sandhu S, Itsiopoulos C. Experiences and perceptions of patients undergoing treatment and quality of life impact of diabetic macular edema: A systematic review. *Psychol , Health Med*. 2019;24(4):383-401. https://doi.org/10.1080/13548506.2018.1533249. doi: 10.1080/13548506.2018.1533249.

41. Taylor DJ, Hobby AE, Binns AM, Crabb DP. How does age-related macular degeneration affect real-world visual ability and quality of life? A systematic review. *BMJ Open*. 2016;6(12):e011504. http://bmjopen.bmj.com/content/6/12/e011504.abstract. doi: 10.1136/bmjopen-2016-011504.

42. Aromataris E, Munn Z, eds. *Joanna briggs institute reviewer's manual.* The Joanna Briggs Institute; 2017.

For peer review only

43. Moher D, Shamseer L, Clarke M, et al. Preferred reporting items for systematic review and meta-analysis protocols (PRISMA-P) 2015 statement. *Systematic Reviews*. 2015;4(1):1. https://doi.org/10.1186/2046-4053-4-1. doi: 10.1186/2046-4053-4-1.

44. WHO | priority eye diseases. WHO Web site. http://www.who.int/blindness/causes/priority/en/. Accessed Jan 21, 2020.

45. Krnic Martinic M, Pieper D, Glatt A, Puljak L. Definition of a systematic review used in overviews of systematic reviews, meta-epidemiological studies and textbooks. *BMC Medical Research Methodology*. 2019;19(1):203. https://doi.org/10.1186/s12874-019-0855-0. doi: 10.1186/s12874-019-0855-0.

46. Aromataris E, Fernandez R, Godfrey CM, Holly C, Khalil H, Tungpunkom P. Summarizing systematic reviews: Methodological development, conduct and reporting of an umbrella review approach. *Int J Evid Based Healthc*. 2015;13(3):132-140. doi: 10.1097/XEB.0000000000000055 [doi].

47. Guyatt GH, Oxman AD, Vist GE, et al. GRADE: An emerging consensus on rating quality of evidence and strength of recommendations. *BMJ*. 2008;336(7650):924-926. http://www.bmj.com/content/336/7650/924.abstract. doi: 10.1136/bmj.39489.470347.AD.

For peer review only

Figure 1 - Organization of Findings

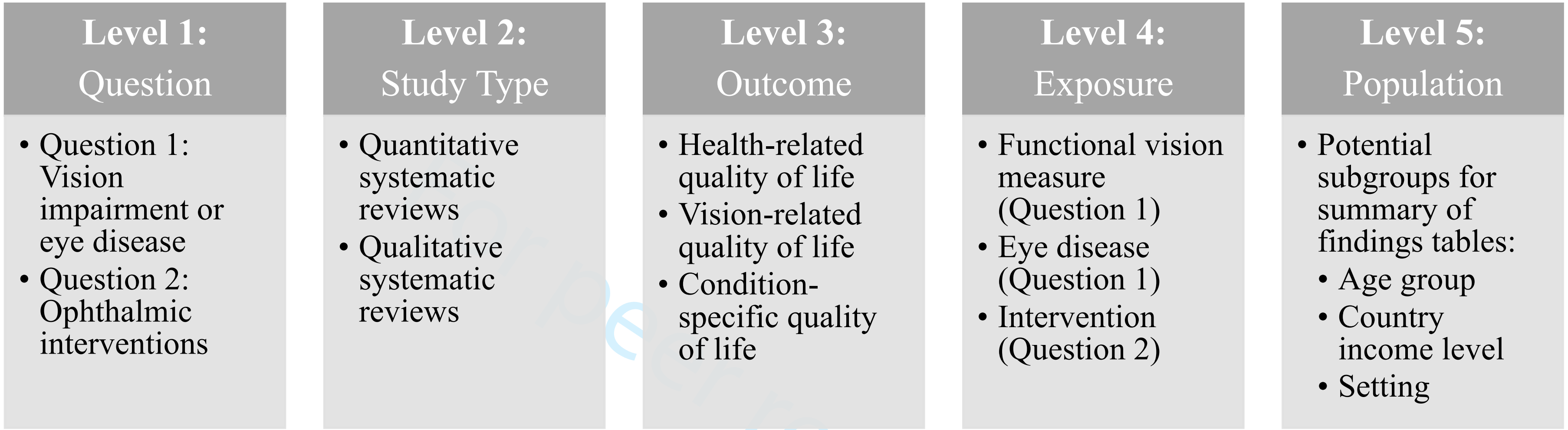

For peer review only

Online Supplementary File 1

Reporting checklist for protocol of a systematic review
Based on the PRISMA-P guidelines.

		Reporting Item	Page Number
**Title**			
Identification	#1a	Identify the report as a protocol of a systematic review	1
Update	#1b	If the protocol is for an update of a previous systematic review, identify as such	NA
**Registration**			
	#2	If registered, provide the name of the registry (such as PROSPERO) and registration number	6
**Authors**			
Contact	#3a	Provide name, institutional affiliation, e-mail address of all protocol authors; provide physical mailing address of corresponding author	1
Contribution	#3b	Describe contributions of protocol authors and identify the guarantor of the review	11-12
**Amendments**			
	#4	If the protocol represents an amendment of a previously completed or published protocol, identify as such and list changes; otherwise, state plan for documenting important protocol amendments	6

For peer review only

**Support**			
Sources	#5a	Indicate sources of financial or other support for the review	12
Sponsor	#5b	Provide name for the review funder and / or sponsor	12
Role of sponsor or funder	#5c	Describe roles of funder(s), sponsor(s), and / or institution(s), if any, in developing the protocol	12
**Introduction**			
Rationale	#6	Describe the rationale for the review in the context of what is already known	5
Objectives	#7	Provide an explicit statement of the question(s) the review will address with reference to participants, interventions, comparators, and outcomes (PICO)	5
**Methods**			
Eligibility criteria	#8	Specify the study characteristics (such as PICO, study design, setting, time frame) and report characteristics (such as years considered, language, publication status) to be used as criteria for eligibility for the review	6-7
Information sources	#9	Describe all intended information sources (such as electronic databases, contact with study authors, trial registers or other grey literature sources) with planned dates of coverage	7-8
Search strategy	#10	Present draft of search strategy to be used for at least one electronic database, including planned limits, such that it could be repeated	7

For peer review only

Study records - data management	#11a	Describe the mechanism(s) that will be used to manage records and data throughout the review	8
Study records - selection process	#11b	State the process that will be used for selecting studies (such as two independent reviewers) through each phase of the review (that is, screening, eligibility and inclusion in meta-analysis)	8-9
Study records - data collection process	#11c	Describe planned method of extracting data from reports (such as piloting forms, done independently, in duplicate), any processes for obtaining and confirming data from investigators	9-10
Data items	#12	List and define all variables for which data will be sought (such as PICO items, funding sources), any pre-planned data assumptions and simplifications	9-10
Outcomes and prioritization	#13	List and define all outcomes for which data will be sought, including prioritization of main and additional outcomes, with rationale	9-10
Risk of bias in individual studies	#14	Describe anticipated methods for assessing risk of bias of individual studies, including whether this will be done at the outcome or study level, or both; state how this information will be used in data synthesis	9-11
Data synthesis	#15a	Describe criteria under which study data will be quantitatively synthesised	NA
Data synthesis	#15b	If data are appropriate for quantitative synthesis, describe planned summary measures, methods of handling data and	NA

For peer review only

		methods of combining data from studies, including any planned exploration of consistency (such as I2, Kendall's τ)	
Data synthesis	#15c	Describe any proposed additional analyses (such as sensitivity or subgroup analyses, meta-regression)	NA
Data synthesis	#15d	If quantitative synthesis is not appropriate, describe the type of summary planned	10-11
Meta-bias(es)	#16	Specify any planned assessment of meta-bias(es) (such as publication bias across studies, selective reporting within studies)	NA
Confidence in cumulative evidence	#17	Describe how the strength of the body of evidence will be assessed (such as GRADE)	11

The PRISMA-P checklist is distributed under the terms of the Creative Commons Attribution License CC-BY 4.0. This checklist was completed on 06. February 2020 using https://www.goodreports.org/, a tool made by the EQUATOR Network in collaboration with Penelope.ai

For peer review only

Online Supplementary File 2

Search strategy for MEDLINE Ovid

1. exp Eye Diseases/

2. exp Eye Injuries/

3. exp Administration, Ophthalmic/

4. exp Diagnostic Techniques, Ophthalmological/

5. exp Eye Protective Devices/

6. exp Glaucoma Drainage Implants/

7. exp Injections, Intraocular/

8. exp Ophthalmic Solutions/

9. exp Ophthalmologic Surgical Procedures/

10. exp Optical Devices/

11. exp Orbital Implants/

12. exp Orthoptics/

13. exp Pseudophakia/

14. exp Visual Prosthesis/

15. ((low* or handicap* or subnormal* or impair* or partial* or disab* or reduce* or diminish* or decrease*) adj3 (vision or visual* or sight*)).tw.

16. ((abnormal* or blurred or defect* or difficult* or dim or disturbed or hazy or interference or poor or tunnel or weak* or defect* or deficienc* or disorder* or disturb* or problem*) adj3 (vision or visual* or sight*)).tw.

17. ((delayed or agnosia or constriction* or prosthesis or prostheses) adj3 (vision or visual* or sight*)).tw.

18. ((vision or visual or sight*) adj2 loss).tw.

19. (Ocular or occular or intraocular or ophthalmol* or ophthalmic* or ophthalmop* or optic* or orbital or conjunctival or conjunctivitis or eye or eyes or eyelid* or cataract* or corneal or glaucoma* or lacrimal or lacrimation or macular or retinal or retinitis or retinoblastoma or retinopath* or retrobulbar neuritis or uveal or uveitis or vitrectomy or vitreous detachment* or vitreous haemorrhage* or vitreous hemorrhage* or vitreous membranes or vitreous strands or vitreous prolapse* or vitreous syneresis).tw.

20. (Amblyopia or Ametropia or Anisocoria or Anophthalmia or Anterior Chamber Haemorrhage or Anterior Chamber Hemorrhage or Aphakia or Aqueous Outflow Obstruction or

For peer review only

Asthenopia or Balint's Syndrome or Blepharitis or Blepharospasm or chalazia or chalazion or Chorioretinal Disorder* or Chorioretinitis or Choroid Diseases or Choroidal or Choroiditis or Chromatopsia or Diplopia or Endophthalmitis or Epiphora or Episcleritis or Equatorial Staphyloma or Esotropia or Exophthalmos or Fixed Pupil* or Fuchs endothelial dystrophy or Hemianopia or Hemianopsia or Hepatolenticular Degeneration or Hordeola or Hordeolum or Horner's Syndrome or Hypopyon or Iritis or Keratitis or Keratoconjunctivitis or Keratoconus or Lens Disease* or Lens Disorder* or Lens Opacit* or Lens Subluxation or Localized Anterior Staphyloma or Meibomianitis or Miosis or Mydriasis or Myopia or Nystagmus or Oculopath* or Papilloedema or Periorbital Fat Herniation or (Periocular and carcinoma*) or Photalgia or Photophobia or Photopsia or Pigment Precipitation or Posterior capsule opacification or Posterior Dislocation Of Lens or Posterior Synechiae or Pseudophakia or Proliferative Vitreoretinopathy or Scleral Disease* or Scleral Staphyloma or Scleritis or Scotoma or Staphyloma Posticum or Strabismus or Symblepharon or Traumatic Hyphema or Wavefront Aberration* or Wegener's granulomatosis or Wilson's Disease or Xerophthalmia or refractive error* or near-sighted* or nearsighted* or short-sighted* or shortsighted* or hyperopia or farsighted* or far sighted* or long-sighted* or longsighted*or astigmatism or presbyopia* or onchocerciasis or onchocerciases).tw.

21. (LASIK or LASEK or Orthoptic* or "visual prosthesis" or "visual prostheses" or "artificial iris" or "capsular tension ring" or "cornea implant" or "intravitreal implant" or "lens implant" or "palpebral spring" or "punctal plug" or "retinal implant" or "sclerectomy implant" or glasses or spectacle* or "artificial lens" or "artificial implant lens" or pseudophakos or "orbit implant" or "ab interno gel implant" or "ab interno gel stent" or "anterior chamber drainage tube" or "aqueous drainage device" or "aqueous drainage implant" or "aqueous shunt" or glaukos or istent or keratoprosthesis).tw.

22. or/1-21

23. "Quality of Life"/ or Quality-Adjusted Life Years/ or "Value of Life"/ or Health Status/ or Sickness Impact Profile/ or Disability Evaluation/ or exp "Activities of Daily Living"/ or Cost-Benefit Analysis/ or "Surveys and Questionnaires"/ or Health surveys/ or exp psychometrics/

24. (quality adj2 life).tw.

25. ("disability adjusted life" or qaly* or qald* or qale* or qtime* or daly* or euroqol or "euro qol" or eq5d or "eq 5d" or hql or hqol or "h qol" or hrqol or "hr qol" or hye or hyes or health* year* equivalent* or hui or hui1 or hui2 or hui3 or "willingness to pay" or "standard gamble" or QOL or HRQL or HRQOL or wellbeing or "well being" or WHOQOL or "healthy days measures" or "EQ VAS" or "EQ 15D" or "36 Item Short Form Survey" or "SF 36" or "12 item Short Form Survey" or "SF 12" or "Visual Function Questionnaire" or "NEI VFQ" or "VFQ 25" or "IND VFQ 33" or "14 item Visual Functioning" or "VF 14" or "11 item Visual Functioning" or "VF 11" or "Impact of Vision Impairment" or IVI or "glaucoma utility index" or catquest or "Activities of Daily Vision Scale" or ADVS or "Cataract Symptom Scale" or "Daily Living Tasks Dependent Upon Vision" or DLTV or "Measure of Outcome in Ocular Disease" or "Refractive Status and Vision Profile" or "Vision Specific Sickness Impact Profile" or SIPV or

For peer review only

"Visual Activities Questionnaire*" or VAQ or "Visual Disability Assessment*" or VDA or "Visual Disabilities Questionnaire*" or "Visual Function Questionnaire*" OR "VA LV VFQ" or "Glaucoma symptom scale" or "Symptom Impact Glaucoma Score" or GHPI or "Glaucoma Health Perceptions index").tw.

26. (health adj3 (utility* or disutili* or state or status)).tw.

27. ((visual or vision) adj3 (disabilit* or disabled or function* or activit* or task or performance or impairment or Questionnaire*)).tw.

28. or/23-27

29. 22 and 28

30. Cochrane Database Syst Rev.ja. or Meta-Analysis.pt. or (Search* or Medline or (Systematic and Review)).tw.

31. limit 29 to systematic reviews

32. 30 or 31

33. exp Animals/ not exp Humans/

34. 32 not 33

35. 29 and 34

For peer review only

BMJ Open

Eye Health and Quality of Life: An Umbrella Review Protocol

Journal:	*BMJ Open*
Manuscript ID	bmjopen-2020-037648.R1
Article Type:	Protocol
Date Submitted by the Author:	10-Jun-2020
Complete List of Authors:	Assi, Lama; Johns Hopkins University School of Medicine, Wilmer Eye Institute<br>Rosman, Lori ; Johns Hopkins University School of Medicine, Welch Medical Library<br>Chamseddine, Fatimah; American University of Beirut Faculty of Medicine, Clinical Research Institute<br>Ibrahim, Perla; American University of Beirut Faculty of Medicine, Department of Ophthalmology<br>Sabbagh, Hadi; American University of Beirut Faculty of Medicine, Department of Ophthalmology<br>Congdon, Nathan; Queen's University Belfast School of Medicine Dentistry and Biomedical Sciences, Centre for Public Health; Sun Yat-Sen University, Zhongshan Ophthalmic Center<br>Evans, Jennifer; London School of Hygiene & Tropical Medicine, International Centre for Eye Health<br>Ramke, Jacqueline; London School of Hygiene & Tropical Medicine, International Centre for Eye Health; The University of Auckland, School of Optometry and Vision Science<br>Kuper, Hannah; London School of Hygiene & Tropical Medicine, International Centre for Evidence in Disability<br>Burton, Matthew J; London School of Hygiene & Tropical Medicine, International Centre for Eye Health; Moorfields Eye Hospital<br>Ehrlich, Joshua; University of Michigan, Department of Ophthalmology and Visual Sciences; University of Michigan, Institute for Healthcare Policy and Innovation<br>Swenor, B; Johns Hopkins University School of Medicine, Wilmer Eye Institute; Johns Hopkins University Bloomberg School of Public Health, Department of Epidemiology
**Primary Subject Heading**:	Ophthalmology
Secondary Subject Heading:	Public health, Global health
Keywords:	OPHTHALMOLOGY, PUBLIC HEALTH, EPIDEMIOLOGY

SCHOLARONE™
Manuscripts

I, the Submitting Author has the right to grant and does grant on behalf of all authors of the Work (as defined in the below author licence), an exclusive licence and/or a non-exclusive licence for contributions from authors who are: i) UK Crown employees; ii) where BMJ has agreed a CC-BY licence shall apply, and/or iii) in accordance with the terms applicable for US Federal Government officers or employees acting as part of their official duties; on a worldwide, perpetual, irrevocable, royalty-free basis to BMJ Publishing Group Ltd ("BMJ") its licensees and where the relevant Journal is co-owned by BMJ to the co-owners of the Journal, to publish the Work in this journal and any other BMJ products and to exploit all rights, as set out in our licence.

The Submitting Author accepts and understands that any supply made under these terms is made by BMJ to the Submitting Author unless you are acting as an employee on behalf of your employer or a postgraduate student of an affiliated institution which is paying any applicable article publishing charge ("APC") for Open Access articles. Where the Submitting Author wishes to make the Work available on an Open Access basis (and intends to pay the relevant APC), the terms of reuse of such Open Access shall be governed by a Creative Commons licence – details of these licences and which Creative Commons licence will apply to this Work are set out in our licence referred to above.

Other than as permitted in any relevant BMJ Author's Self Archiving Policies, I confirm this Work has not been accepted for publication elsewhere, is not being considered for publication elsewhere and does not duplicate material already published. I confirm all authors consent to publication of this Work and authorise the granting of this licence.

For peer review only

Eye Health and Quality of Life: An Umbrella Review Protocol

Lama Assi[1], MD, Lori Rosman[2], MLS, Fatimah Chamseddine[3], MD, Perla Ibrahim[4], MD, Hadi Sabbagh[4], BS, Nathan Congdon[5,6,7], MD, MPH, Jennifer Evans[8], MSc, PhD, Jacqueline Ramke[8,9], PhD, MPH, Hannah Kuper[10], ScD, Matthew J Burton[8,11], PhD, FRCOphth, Joshua R Ehrlich[12,13], MD, MPH, Bonnielin K Swenor[1,14]*, PhD, MPH

Author Affiliations

1. Wilmer Eye Institute, Johns Hopkins University School of Medicine, Baltimore, USA
2. Welch Medical Library, Johns Hopkins University School of Medicine, Baltimore, USA
3. Clinical Research Institute, American University of Beirut Faculty of Medicine, Beirut, Lebanon
4. Department of Ophthalmology, American University of Beirut Faculty of Medicine, Beirut, Lebanon
5. Centre for Public Health, Queen's University Belfast School of Medicine Dentistry and Biomedical Sciences, Belfast, UK
6. Orbis International, New York, United States
7. Zhongshan Ophthalmic Center, Sun Yat-sen University, Guangzhou, China
8. International Centre for Eye Health, London School of Hygiene & Tropical Medicine, London, UK
9. School of Optometry and Vision Science, University of Auckland, Auckland, New Zealand
10. International Centre for Evidence in Disability, London School of Hygiene & Tropical Medicine, London, UK
11. Moorfields Eye Hospital, London, UK
12. Department of Ophthalmology and Visual Sciences, University of Michigan, Ann Arbor, MI, USA
13. Institute for Healthcare Policy and Innovation, University of Michigan, Ann Arbor, MI, USA
14. Department of Epidemiology, Johns Hopkins Bloomberg School of Public Health, Baltimore, MD, USA

*Corresponding author:
Bonnielin K Swenor
bswenor@jhmi.edu
600 N. Wolfe Street
Wilmer 116
Baltimore, MD 21287
Phone: 410-614-2874

Word count: 2865

For peer review only

ABSTRACT

Introduction: Vision impairment and eye disease are major global health concerns and have been associated with increased morbidity and mortality, and lower quality of life. Quality of life, whether generic, vision-specific, or disease-specific, is an important measure of the impact of eye health on people's daily activities, well-being, and visual function, and is increasingly used to evaluate the impact of ophthalmic interventions and new devices. While many studies and reviews have examined the relationship between vision or eye health and quality of life across different contexts, there has yet to be a synthesis of the impact of vision impairment, eye disease, and ophthalmic interventions on quality of life globally and across the lifespan.

Methods and analysis: An umbrella review of systematic reviews will be conducted to address these two questions: 1) What is the association of vision impairment and eye disease with quality of life; and 2) What is the impact of ophthalmic interventions on quality of life? A search of related literature will be performed on the 11th February 2020 in Medline Ovid, Embase.com, Cochrane Database of Systematic Reviews, Proquest Dissertations and Theses Global, and the grey literature, and repeated at the synthesis stage. Title/abstract and full-text screening, methodological quality assessment, and data extraction will be conducted by reviewers working independently and in duplicate. Assessment of methodological quality and data extraction will be performed using Joanna Briggs Institute standard forms. Findings from the systematic reviews and their methodological quality will be summarized qualitatively in the text and using tables.

Ethics and dissemination: No ethical approval is required. Results of this umbrella review will be published in a peer-reviewed journal and summarized in the *Lancet Global Health* Commission on Global Eye Health.

Registration: This protocol was registered in the Open Science Framework Registries (https://osf.io/qhv9g/).

Keywords: ophthalmology, public health, epidemiology

For peer review only

ARTICLE SUMMARY

Strengths and limitations of this study:

- The umbrella review approach allows for a comprehensive review of a very broad topic by summarizing the evidence from multiple research syntheses into one systematic review of reviews.
- Study screening, critical appraisal, and data collection will be conducted in duplicate.
- Standardized forms developed specifically for the conduct of umbrella reviews will be used for critical appraisal and data collection.
- Studies related to rare topics or special settings might not be included in systematic reviews, and thus would not be represented in this umbrella review, a main limitation of our work.
- Only systematic reviews published in English will be included.

For peer review only

INTRODUCTION

Vision impairment is a major cause of disability worldwide.[1] In 2015, an estimated 36 million people were blind, 217 million had moderate or severe vision impairment, and over a billion people experienced near-vision impairment (presbyopia).[2] Cataract and uncorrected refractive error are correctable conditions which accounted for 78% of global visual impairment that year.[3] Despite reductions in age-specific prevalence, the number of people with vision impairment and blindness is projected to increase due to population growth and aging.[2] Vision impairment is associated with negative health outcomes, such as having multiple chronic conditions,[4,5] and increased mortality,[6] and also induces substantial socioeconomic consequences for individuals,[7] and an associated lower quality of life.

Objective clinical measures, like visual acuity, intraocular pressure, or fundus imaging, are widely used in the clinical and research settings to assess eye health, but often fail to capture the impact of vision impairment or eye disease on individuals' daily activities or social well-being.[8] Quality of life instruments, on the other hand, measure patient-reported outcomes, such as perceived health, physical, mental, emotional, or social well-being, and even vision-specific function. These measures are important as vision impairment can have a large impact on quality of life, possibly to an even greater degree than major conditions such as stroke, heart disease, or diabetes.[9] Both severe conditions that lead to marked reduction in vision like age-related macular degeneration,[10] and highly symptomatic conditions which may not be associated with impaired vision, like dry eye syndrome,[11] have been associated with decreased health-related quality of life.

The use of quality of life instruments has gained popularity in ophthalmic studies, including clinical trials, over the past decade.[12] While there is a wide range of quality of life instruments available, vision-related quality of life instruments are frequently used in ophthalmic studies, as these questionnaires are more sensitive to the impact of subtle vision changes on daily function compared to more general health-related or generic quality of life tools.[13] Reduced visual acuity[14] and visual field loss[15] are both associated with worsening in vision-related quality of life; and glaucoma[16-19] and cataract[16,19] are associated with worse vision-related function, independent of visual acuity. In ophthalmic clinical trials, health-related, vision-related, and even disease-specific scales have been used as secondary outcome measures, and more recently, as primary outcomes as well.[20,21] Patient-reported outcomes are also increasingly being incorporated in the evaluation of new ophthalmic devices, and the

For peer review only

Food and Drug Administration even provides guidance on using them to support labeling claims too.[22,23]

There has yet to be a global assessment of the impact of eye health, including vision impairment, eye disease, and ophthalmic interventions on quality of life across the lifespan, despite the growing number of ophthalmic studies assessing quality of life, and increased value placed on patient-reported outcomes. Prior studies on vision and quality of life have usually focused on specific countries (e.g., USA,[24] Finland,[25] South Korea,[26] Nigeria[27]), populations (e.g., Malay population in Singapore,[28] Latino population[29] and indigenous peoples of the Americas[30] in the USA), or settings (e.g., community,[31] outpatient clinics[32]). Even reviews summarizing the evidence about the impact of vision on quality of life have usually focused on specific age groups (e.g., children,[33,34] older adults[35]), eye conditions (e.g., glaucoma,[36] diabetic retinopathy,[37] dry eye[38]), or interventions (e.g., low vision rehabilitation for children,[34] anti-VEGF treatment for age-related macular degeneration[39]).

This umbrella review (or systematic review of systematic reviews) will examine the impact of vision impairment, eye disease and ophthalmic interventions on quality of life globally and across the lifespan. An umbrella review approach allows us to maintain a broad scope while relying on the highest quality of evidence, given the large number of primary studies[9-11,14-19,24-32] and reviews on this topic[34-41]. A search of three systematic review registries (PROSPERO, Joanna Briggs Institute Systematic Review Register, and Open Science Framework Registries) has shown that there is currently no systematic or umbrella review underway for this topic.

Objectives and Questions

This umbrella review of systematic reviews aims to identify and synthesize currently available knowledge about the association of vision and eye disease with quality of life on a global level. Two questions will be addressed:

1) What is the association between vision impairment or eye disease and quality of life?
2) What is the impact of ophthalmic interventions on quality of life?

For peer review only

METHODS AND ANALYSIS

This protocol was registered in the Open Science Framework Registries (https://osf.io/qhv9g/). It was designed by following the Joanna Briggs Institute guidelines for the conduct and preparation of umbrella reviews,[42] and the Preferred Reporting Items for Systematic review and Meta-Analysis Protocols (PRISMA-P) guidelines for the reporting of systematic review protocols (online supplementary file 1).[43] The anticipated start date of this study is the 11th February 2020. Any changes to the methodological approach will be dated and described in detail in the final umbrella review report.

Inclusion Criteria

Systematic reviews and meta-analyses that evaluate the impact of vision impairment, eye disease, or ophthalmic interventions on quality of life will be included in this umbrella review.

Types of participants

Systematic reviews of studies with participants who have vision impairment, or an eye disease will be included. Vision impairment can be self-reported or assessed objectively, using any measure of visual function, including, but not limited to, visual acuity (corrected or uncorrected, distance or near), contrast sensitivity, or visual fields. Eye disease diagnosis can be based on self-report, medical chart or claims data, or an objective assessment of symptoms, clinical signs, or imaging findings. Eye diseases that will be explored include, but are not limited to, the World Health Organization (WHO) priority eye diseases, which are the most common causes of vision impairment worldwide: cataract, onchocerciasis, trachoma, refractive errors, age-related macular degeneration, diabetic retinopathy, glaucoma, corneal opacities, childhood blindness, and genetic eye diseases.[44]

Systematic reviews with sample populations from any age group (children, working-age adults, or older adults), country (low, middle or high income), or setting (community, hospital, clinic, institution) will be included.

Interventions

Systematic reviews that examine interventions will also be included and will help answer the second question specifically, the impact of ophthalmic interventions on quality of life. The

For peer review only

comparison group can be the same group pre-intervention, another group that receives another intervention, or another group receiving no intervention. Any ophthalmic intervention identified will be included, as long as its main aim is to correct or improve vision, slow down the progression of vision loss, improve functional ability among those with vision loss (e.g., low vision rehabilitation, use of assistive devices), or relieve eye pain or discomfort.

Outcomes

Studies that measure any aspect of quality of life (generic or health-related, vision-related, or disease-specific) will be included. Studies can report on one or all domains used to measure quality of life. Systematic reviews of both quantitative and qualitative studies are eligible for inclusion. Examples of quality of life instruments are the WHO Quality of Life Assessment Instrument (health-related quality of life), the National Eye Institute Vision Function Questionnaire (vision-related quality of life), and the Catquest-9SF questionnaire (cataract-specific quality of life).

Types of studies

Only systematic reviews (with or without and meta-analyses) are eligible for inclusion. A systematic review will be defined as a review that includes every one of these items: a research question, a search strategy with the sources searched, inclusion and exclusion criteria, screening methods, a discussion about the quality of included studies and risk of bias, and information about data analysis and synthesis.[45] Systematic reviews of observational and interventional studies will be included, but those that incorporate case series or expert opinion as their source of evidence will be excluded. All other types of reviews, including narrative reviews and scoping reviews will be excluded.

Search Strategy

An academic librarian developed a comprehensive search strategy based on similar ones used by Cochrane Eyes and Vision Group. Search strategies were developed using a combination of controlled vocabulary and keywords to represent vision terms, eye diseases, including all the WHO priority eye disease listed above, and ophthalmic interventions, as well as search terms for quality of life, including some commonly used scales, and terms to identify systematic reviews (see Online Supplementary File 2 for a detailed search strategy).

For peer review only

The following databases will be searched on the 11th February 2020: Medline Ovid (1946 to present), Embase.com (1947 to present), Cochrane Database of Systematic Reviews (1995 to present), Proquest Dissertations and Theses Global (1861 to present). A search for grey literature will include sources such as reports from governments and non-governmental organizations, and databases including the Open Grey and the Agency for Healthcare Research and Quality. The search will be limited to articles published in English with no restrictions on the year of publication. We will search the references of included studies for additional systematic reviews.

The search will be run again in the synthesis stage to identify any relevant reviews published since the initial search.

Study Selection

Citations retrieved from the searches will be imported to Endnote, where any duplicates will be removed. Then, references will be imported to Covidence, a web-based software platform that streamlines the production of systematic reviews. Four reviewers will work in pairs to screen the studies independently and in duplicate. Conflicts will be discussed in the presence of a third reviewer from the second pair; if no consensus is reached, the senior author will be consulted.

Review selection will take place at the level of title/abstract and full text. Reviews judged to be of insufficient quality for inclusion will also be excluded at the methodological assessment stage. Articles will first be screened at the level of the titles and abstracts. At this stage, all systematic reviews and meta-analyses, published in English, and that address a vision-related topic (vision impairment, eye disease, or ophthalmic intervention) and quality of life will be included. Articles that are identified as systematic reviews in the title or abstract, using the terms “systematic review” or “meta-analysis”, will be included. Reviews that are not explicitly identified as such will be moved to full-text review if the methods suggest they may be systematic reviews based on the definition used above. Reviews that are clearly not related to quality of life will be excluded at this stage, but reviews of interventional studies that do not specifically address quality of life in their title or abstract will go to full-text review, as quality of life may be a secondary outcome that is only mentioned in the text.

For peer review only

In the next step, the included reviews will undergo full-text screening and will be included if they meet the criteria listed above for a systematic review, if none of their primary studies are case series/case reports or expert opinion, and if they specifically assess the impact of the vision-related topic on quality of life. The final study selection will be made after assessment of methodological quality. Reasons for exclusion will be logged.

Assessment of Methodological Quality

Systematic reviews that are deemed eligible for inclusion will be assessed for their methodological quality using the JBI Critical Appraisal Checklist for Systematic Reviews and Research Syntheses.[46] The four reviewers will work again in pairs to do the assessment independently and in duplicate. JBI SUMARI, a web-based review software that has partnered with Covidence, will be used to facilitate the critical appraisal step.

The JBI Critical Appraisal Checklist contains 11 items related to systematic review methodology, each graded as "Yes", "No", "Unclear", or "Not Applicable". It can be used to appraise both quantitative and qualitative systematic reviews. The form will be piloted by the four reviewers by testing it on two studies before starting independent appraisals; these reviewers will compare their results and discuss what constitutes an acceptable level of information to decide if a review meets or does not meet the criteria, and when is it "Unclear". Systematic reviews for which at least one of the items "clear review question", "appropriate inclusion criteria", "appropriate search strategy", or "appropriate criteria for critical appraisal" are graded as "Unclear" or "No" will be considered to be of insufficient quality for inclusion, and as such would be excluded at this stage.

Results of the quality appraisal for each review will be presented in a table, and the overall methodological quality of the included reviews will be summarized in the text.

Data Collection

Data will be extracted from the final list of articles included using the JBI Data Extraction Form for Review for Systematic Reviews and Research Syntheses (see Online Supplementary File 3 for a blank copy of the sample data extraction sheet).[42] Again, the four

For peer review only

reviewers will work in pairs to extract the data independently and in duplicate, using the JBI SUMARI software.

In brief, the standardized form will be used to extract information about citation details (e.g., author, year), systematic review methodology (e.g., objectives, participants, setting/context, search strategy, appraisal instrument used), characteristics of the included studies (e.g., date range, number and types of studies, country of origin, rating of their quality, outcomes reported), and findings of the systematic review (e.g., method of synthesis/analysis employed, results/findings). Additionally, information about the review's funder or sponsor and their role, when applicable, and the reviewers' overall assessment of the quality of the evidence, such as GRADE (Grading of Recommendations Assessment, Development, and Evaluation), will be collected. The GRADE quality assessment is based on the primary studies' quality and design, and the consistency and directness of the findings.[47]

In the comments section, the following information will be indicated: 1) if the review is about vision impairment/eye health or an ophthalmic intervention, 2) the functional vision measure used or eye disease or intervention, 3) if the population belongs to a specific age group, country income group, or setting. This will help classify the reviews for the synthesis.

Before the reviewers start collecting data independently, the form will be piloted. All four reviewers will extract data from two articles, compare their answers and discuss them to ensure that they all interpret the questions in the same way.

Data Summary

A qualitative synthesis of the findings will be presented in the text and using tables describing study characteristics and the overall umbrella review results (summary of findings). When presenting study characteristics, studies will first be divided according to the question they address: the impact of vision impairment or eye disease on quality of life, or the impact of ophthalmic interventions on quality of life. Each question will have a table for the quantitative systematic reviews, and another one for the qualitative systematic reviews, as the study characteristic information reported/presented for each are different. Within each table, results will be stratified according to the outcome measured (health-related, vision-related, or

For peer review only

condition-specific quality of life), and functional vision measured, eye disease, or intervention (Figure 1).

Study characteristics tables for the quantitative systematic reviews will include the following information: the number of studies in the systematic review, the number of participants from the included studies, estimates computed, and the heterogeneity of the results. For qualitative systematic reviews, the final synthesized findings will be presented along with information about the study context. Overlaps of original research studies in the included systematic reviews will be presented.

Summary of findings tables will present an overall summary for each question, exposure, outcome, and, when applicable, subgroup (age, country income group, setting) (Figure 1), along with an assessment of the strength of the evidence for each finding, such as GRADE, when included in the review.

Study Limitations

The study methodology has some limitations that may impact the final results of the review. Using the umbrella review approach limits the results to those found in articles that have been included in systematic reviews. While this means that studies about rare diseases or topics that have not yet been addressed by systematic reviews will not be included, it is this approach that will allow us to perform a global assessment of a broad topic in a systematic manner. Moreover, using strict criteria to define a systematic review, and limiting inclusion to those that meet certain quality requirements may further decrease the number of studies included, but it will allow us to focus on the available high-quality evidence. In regard to the first question, vision impairment and eye diseases may be defined and diagnosed differently in each review, thus making it harder to combine the evidence. Likewise, a large number of interventions may be identified for the second question, and the type of comparison groups might differ between reviews (intervention compared to no intervention, or intervention compared to another intervention), making the synthesis of the evidence challenging. However, using the methods detailed above to present the results and summarize the findings will allow us to organize the findings in a systematic manner and present enough context for the reader to interpret the results. Finally, as with any umbrella review, there may be overlap in the primary studies included in each systematic review; however, we will highlight any overlap of studies in the tables.

For peer review only

Patient and Public Involvement

There is no patient or public involvement in this study.

ETHICS AND DISSEMINATION

Only published studies will be examined for this systematic review; therefore, no ethical approval is required. If any changes to the protocol are made, they will be described in the final umbrella review report.

Results from this study will be published in a peer-reviewed journal and summarized in *The Lancet Global Health* Commission on Global Eye Health.

Figure Legends

Figure 1: Organization of Findings. Reviews will be divided according to the question they address (question 1 being vision impairment/eye disease, and question 2, ophthalmic interventions). For each question, reviews will be categorized as quantitative or qualitative, and within each category, they will be further grouped based on their quality of life measure and exposure (specific functional vision measure or eye disease for question 1, and intervention for question 2). Summary of findings tables will be further stratified by study population depending on the results available; potential subgroups include age category (children, working-age adults, or older adults), country (low, middle or high income), or setting (community, hospital, clinic, institution).

Acknowledgments: None.

Author Contributions: BKS is the guarantor of the review. BKS, LA, LR, FC, PI, HS, NC, JRE, JR, HK, MJB, and JE contributed to the development of the selection criteria, the risk of bias assessment strategy and data extraction criteria. LR developed the search strategy. NC, JRE, JR, HK, MJB, and JE provided expertise on systematic and umbrella review methodology, and global eye health. LA, LR and BKS drafted the manuscript. FC, PI, HS, NC, JRE, JR, HK, MJB, and JE revised the manuscript critically for important intellectual content. BKS, LA, LR, FC, PI, HS, NC, JRE, JR, HK, MJB, and JE approved the final manuscript.

For peer review only

Funding Statement: BKS is supported by the National Institutes of Health (NIA K01AG052640). MJB is supported by the Wellcome Trust (207472/Z/17/Z). JR is a Commonwealth Rutherford Fellow, funded by the UK government through the Commonwealth Scholarship Commission in the UK. JRE is supported by the National Institutes of Health (K23EY027848). The Lancet Global Health Commission on Global Eye Health is supported by The Queen Elizabeth Diamond Jubilee Trust, Moorfields Eye Charity [grant number GR001061], NIHR Moorfields Biomedical Research Centre, The Wellcome Trust, Sightsavers, The Fred Hollows Foundation, The SEVA Foundation, The British Council for the Prevention of Blindness and Christian Blind Mission. No funder had any role in the design or conduct of this work.

Competing Interests Statement: All authors declare that they have no conflict of interest.

For peer review only

REFERENCES

1. GBD 2017 Disease and Injury Incidence and Prevalence Collaborators. Global, regional, and national incidence, prevalence, and years lived with disability for 354 diseases and injuries for 195 countries and territories, 1990-2017: A systematic analysis for the global burden of disease study 2017. *Lancet*. 2018;392(10159):1789-1858. doi: S0140-6736(18)32279-7 [pii].

2. Bourne RRA, Flaxman SR, Braithwaite T, et al. Magnitude, temporal trends, and projections of the global prevalence of blindness and distance and near vision impairment: A systematic review and meta-analysis. *Lancet Glob Health*. 2017;5(9):e888-e897. doi: S2214-109X(17)30293-0 [pii].

3. Flaxman SR, Bourne RRA, Resnikoff S, et al. Global causes of blindness and distance vision impairment 1990-2020: A systematic review and meta-analysis. *Lancet Glob Health*. 2017;5(12):e1221-e1234. doi: S2214-109X(17)30393-5 [pii].

4. Garin N, Olaya B, Lara E, et al. Visual impairment and multimorbidity in a representative sample of the spanish population. *BMC Public Health*. 2014;14:815-815. doi: 10.1186/1471-2458-14-815 [doi].

5. Court H, McLean G, Guthrie B, Mercer SW, Smith DJ. Visual impairment is associated with physical and mental comorbidities in older adults: A cross-sectional study. *BMC Med*. 2014;12:181-7. doi: 10.1186/s12916-014-0181-7 [doi].

6. Kuper H, Mathenge W, Macleod D, et al. Mortality during 6 years of follow-up in relation to visual impairment and eye disease: Results from a population-based cohort study of people aged 50 years and above in Nakuru, Kenya. *BMJ Open*. 2019;9(6):e029700. http://bmjopen.bmj.com/content/9/6/e029700.abstract. doi: 10.1136/bmjopen-2019-029700.

7. Cumberland PM, Rahi JS, UK Biobank Eye and Vision Consortium. Visual function, social position, and health and life chances: The UK biobank study. *JAMA Ophthalmol*. 2016;134(9):959-966. doi: 10.1001/jamaophthalmol.2016.1778 [doi].

8. Denniston AK, Kyte D, Calvert M, Burr JM. An introduction to patient-reported outcome measures in ophthalmic research. *Eye*. 2014;28(6):637-645. https://doi.org/10.1038/eye.2014.41. doi: 10.1038/eye.2014.41.

9. Chia E, Wang JJ, Rochtchina E, Smith W, Cumming RR, Mitchell P. Impact of bilateral visual impairment on health-related quality of life: The Blue Mountains Eye Study. *Invest Ophthalmol Vis Sci*. 2004;45(1):71-76. Accessed Jan 21, 2020. doi: 10.1167/iovs.03-0661.

10. Knudtson MD, Klein BEK, Klein R, Cruickshanks KJ, Lee KE. Age-related eye disease, quality of life, and functional activity. *Arch Ophthalmol*. 2005;123(6):807-814. Accessed Jan 21, 2020. doi: 10.1001/archopht.123.6.807.

11. Paulsen AJ, Cruickshanks KJ, Fischer ME, et al. Dry eye in the Beaver Dam Offspring Study: Prevalence, risk factors, and health-related quality of life. *Am J Ophthalmol*. 2014;157(4):799-806. Accessed Jan 21, 2020. doi: 10.1016/j.ajo.2013.12.023.

For peer review only

12. Braithwaite T, Calvert M, Gray A, Pesudovs K, Denniston AK. The use of patient-reported outcome research in modern ophthalmology: Impact on clinical trials and routine clinical practice. *Patient Relat Outcome Meas*. 2019;10:9-24. Accessed Jan 21, 2020. doi: 10.2147/PROM.S162802.

13. Margolis MK, Coyne K, Kennedy-Martin T, Baker T, Schein O, Revicki DA. Vision-specific instruments for the assessment of health-related quality of life and visual functioning: A literature review. *Pharmacoeconomics*. 2002;20(12):791-812. Accessed Jan 21, 2020. doi: 10.2165/00019053-200220120-00001.

14. McKean-Cowdin R, Varma R, Hays RD, Wu J, Choudhury F, Azen SP. Longitudinal changes in visual acuity and health-related quality of life: The Los Angeles Latino Eye Study. *Ophthalmology*. 2010;117(10):1900-1907, 1907.e1. Accessed Jan 21, 2020. doi: 10.1016/j.ophtha.2010.01.059.

15. Patino CM, Varma R, Azen SP, Conti DV, Nichol MB, McKean-Cowdin R. The impact of change in visual field on health-related quality of life the Los Angeles Latino Eye Study. *Ophthalmology*. 2011;118(7):1310-1317. Accessed Jan 21, 2020. doi: 10.1016/j.ophtha.2010.12.018.

16. Chiang PPC, Zheng Y, Wong TY, Lamoureux EL. Vision impairment and major causes of vision loss impacts on vision-specific functioning independent of socioeconomic factors. *Ophthalmology*. 2013;120(2):415-422. Accessed Jan 21, 2020. doi: 10.1016/j.ophtha.2012.07.077.

17. Lamoureux EL, Chong EW, Thumboo J, et al. Vision impairment, ocular conditions, and vision-specific function: The Singapore Malay Eye Study. *Ophthalmology*. 2008;115(11):1973-1981. Accessed Jan 21, 2020. doi: 10.1016/j.ophtha.2008.05.005.

18. Fenwick EK, Ong PG, Man REK, et al. Vision impairment and major eye diseases reduce vision-specific emotional well-being in a Chinese population. *Br J Ophthalmol*. 2017;101(5):686-690. Accessed Jan 21, 2020. doi: 10.1136/bjophthalmol-2016-308701.

19. Nirmalan PK, Tielsch JM, Katz J, et al. Relationship between vision impairment and eye disease to vision-specific quality of life and function in rural India: The Aravind comprehensive eye survey. *Invest Ophthalmol Vis Sci*. 2005;46(7):2308-2312. Accessed Jan 21, 2020. doi: 10.1167/iovs.04-0830.

20. Lamoureux EL, Mcintosh R, Constantinou M, et al. Comparing the effectiveness of selective laser trabeculoplasty with topical medication as initial treatment (the Glaucoma Initial Treatment Study): Study protocol for a randomised controlled trial. *Trials*. 2015;16:406. https://www.ncbi.nlm.nih.gov/pubmed/26362541 https://www.ncbi.nlm.nih.gov/pmc/articles/PMC4567808/. doi: 10.1186/s13063-015-0924-6.

21. Gazzard G, Konstantakopoulou E, Garway-Heath D, et al. Laser in glaucoma and ocular hypertension (LiGHT) trial. A multicentre, randomised controlled trial: Design and methodology. *Br J Ophthalmol*. 2018;102(5):593-598. Accessed Jan 21, 2020. doi: 10.1136/bjophthalmol-2017-310877.

For peer review only

22. NEI and FDA address the use of patient reported outcomes in medical product development. www.eyeresearch.org Web site. www.eyeresearch.org/events/NEI_FDA_PRO_Symposium. Updated 2013. Accessed February 3, 2020.

23. U.S. Food and Drug Administration. Guidance for industry patient-reported outcome measures: Use in medical product development to support labeling claims. www.fda.gov Web site. https://www.fda.gov/regulatory-information/search-fda-guidance-documents/patient-reported-outcome-measures-use-medical-product-development-support-labeling-claims. Updated 2009. Accessed February 3, 2020.

24. Crews JE, Chou CF, Zack MM, et al. The association of health-related quality of life with severity of visual impairment among people aged 40-64 years: Findings from the 2006-2010 Behavioral Risk Factor Surveillance System. *Ophthalmic Epidemiol*. 2016;23(3):145-153. doi: 10.3109/09286586.2016.1168851 [doi].

25. Taipale J, Mikhailova A, Ojamo M, et al. Low vision status and declining vision decrease health-related quality of life: Results from a nationwide 11-year follow-up study. *Qual Life Res*. 2019;28(12):3225-3236. doi: 10.1007/s11136-019-02260-3 [doi].

26. Park Y, Shin JA, Yang SW, et al. The relationship between visual impairment and health-related quality of life in Korean adults: The Korea National Health and Nutrition Examination survey (2008–2012). *PLOS ONE*. 2015;10(7):e0132779. https://doi.org/10.1371/journal.pone.0132779.

27. Bekibele CO, Gureje O. Impact of self-reported visual impairment on quality of life in the Ibadan Study of Ageing. *Br J Ophthalmol*. 2008;92(5):612-615. doi: 10.1136/bjo.2007.124859 [doi].

28. Tong L, Waduthantri S, Wong TY, et al. Impact of symptomatic dry eye on vision-related daily activities: The Singapore Malay Eye Study. *Eye (Lond)*. 2010;24(9):1486-1491. doi: 10.1038/eye.2010.67 [doi].

29. Sandhu RK, Munoz BE, Swenor BK, West SK. Refractive error and visual function difficulty in a Latino population. *Ophthalmology*. 2012;119(9):1731-1736. doi: 10.1016/j.ophtha.2012.03.003 [doi].

30. McClure TM, Choi D, Becker T, Cioffi GA, Mansberger SL. The effect of visual impairment on vision-related quality of life in American Indian/Alaska natives. *Ophthalmic Epidemiol*. 2009;16(2):128-135. https://doi.org/10.1080/09286580902745428. doi: 10.1080/09286580902745428.

31. Le Q, Zhou X, Ge L, Wu L, Hong J, Xu J. Impact of dry eye syndrome on vision-related quality of life in a non-clinic-based general population. *BMC Ophthalmol*. 2012;12:22-22. doi: 10.1186/1471-2415-12-22 [doi].

32. Adigun K, Oluleye TS, Ladipo MM, Olowookere SA. Quality of life in patients with visual impairment in Ibadan: A clinical study in primary care. *J Multidiscip Healthc*. 2014;7:173-178. doi: 10.2147/JMDH.S51359 [doi].

For peer review only

33. Castañeda YS, Cheng-Patel C, Leske DA, et al. Quality of life and functional vision concerns of children with cataracts and their parents. *Eye*. 2016;30(9):1251-1259. https://www.ncbi.nlm.nih.gov/pubmed/27391939 https://www.ncbi.nlm.nih.gov/pmc/articles/PMC5023803/. doi: 10.1038/eye.2016.134.

34. Elsman EBM, Al Baaj M, van Rens, Gerardus H. M. B., et al. Interventions to improve functioning, participation, and quality of life in children with visual impairment: A systematic review. *Survey of Ophthalmology*. 2019;64(4):512-557. http://www.sciencedirect.com/science/article/pii/S0039625718301516. doi: https://doi.org/10.1016/j.survophthal.2019.01.010.

35. Nyman SR, Dibb B, Victor CR, Gosney MA. Emotional well-being and adjustment to vision loss in later life: A meta-synthesis of qualitative studies. *Disabil Rehabil*. 2012;34(12):971-981. https://doi.org/10.3109/09638288.2011.626487. doi: 10.3109/09638288.2011.626487.

36. Quaranta L, Riva I, Gerardi C, Oddone F, Floriano I, Konstas AGP. Quality of life in glaucoma: A review of the literature. *Adv Ther*. 2016;33(6):959-981. https://doi.org/10.1007/s12325-016-0333-6. doi: 10.1007/s12325-016-0333-6.

37. Khoo K, Man REK, Rees G, Gupta P, Lamoureux EL, Fenwick EK. The relationship between diabetic retinopathy and psychosocial functioning: A systematic review. *Quality of Life Research*. 2019;28(8):2017-2039. https://doi.org/10.1007/s11136-019-02165-1. doi: 10.1007/s11136-019-02165-1.

38. McDonald M, Patel DA, Keith MS, Snedecor SJ. Economic and humanistic burden of dry eye disease in Europe, North America, and Asia: A Systematic literature review. *The Ocular Surface*. 2016;14(2):144-167. http://www.sciencedirect.com/science/article/pii/S1542012415002049. doi: https://doi.org/10.1016/j.jtos.2015.11.002.

39. Boyle J, Vukicevic M, Koklanis K, Itsiopoulos C. Experiences of patients undergoing anti-VEGF treatment for neovascular age-related macular degeneration: A systematic review. *Psychol Health Med*. 2015;20(3):296-310. Accessed Jan 21, 2020. doi: 10.1080/13548506.2014.936886.

40. Rose MA, Vukicevic M, Koklanis K, Rees G, Sandhu S, Itsiopoulos C. Experiences and perceptions of patients undergoing treatment and quality of life impact of diabetic macular edema: A systematic review. *Psychol , Health Med*. 2019;24(4):383-401. https://doi.org/10.1080/13548506.2018.1533249. doi: 10.1080/13548506.2018.1533249.

41. Taylor DJ, Hobby AE, Binns AM, Crabb DP. How does age-related macular degeneration affect real-world visual ability and quality of life? A systematic review. *BMJ Open*. 2016;6(12):e011504. http://bmjopen.bmj.com/content/6/12/e011504.abstract. doi: 10.1136/bmjopen-2016-011504.

42. Aromataris E, Munn Z, eds. *Joanna Briggs Institute Reviewer's Manual.* The Joanna Briggs Institute; 2017.

For peer review only

43. Moher D, Shamseer L, Clarke M, et al. Preferred reporting items for systematic review and meta-analysis protocols (PRISMA-P) 2015 statement. *Systematic Reviews*. 2015;4(1):1. https://doi.org/10.1186/2046-4053-4-1. doi: 10.1186/2046-4053-4-1.

44. WHO | priority eye diseases. WHO Web site. http://www.who.int/blindness/causes/priority/en/. Accessed Jan 21, 2020.

45. Krnic Martinic M, Pieper D, Glatt A, Puljak L. Definition of a systematic review used in overviews of systematic reviews, meta-epidemiological studies and textbooks. *BMC Medical Research Methodology*. 2019;19(1):203. https://doi.org/10.1186/s12874-019-0855-0. doi: 10.1186/s12874-019-0855-0.

46. Aromataris E, Fernandez R, Godfrey CM, Holly C, Khalil H, Tungpunkom P. Summarizing systematic reviews: Methodological development, conduct and reporting of an umbrella review approach. *Int J Evid Based Healthc*. 2015;13(3):132-140. doi: 10.1097/XEB.0000000000000055 [doi].

47. Guyatt GH, Oxman AD, Vist GE, et al. GRADE: An emerging consensus on rating quality of evidence and strength of recommendations. *BMJ*. 2008;336(7650):924-926. http://www.bmj.com/content/336/7650/924.abstract. doi: 10.1136/bmj.39489.470347.AD.

For peer review only

Figure 1 - Organization of Findings

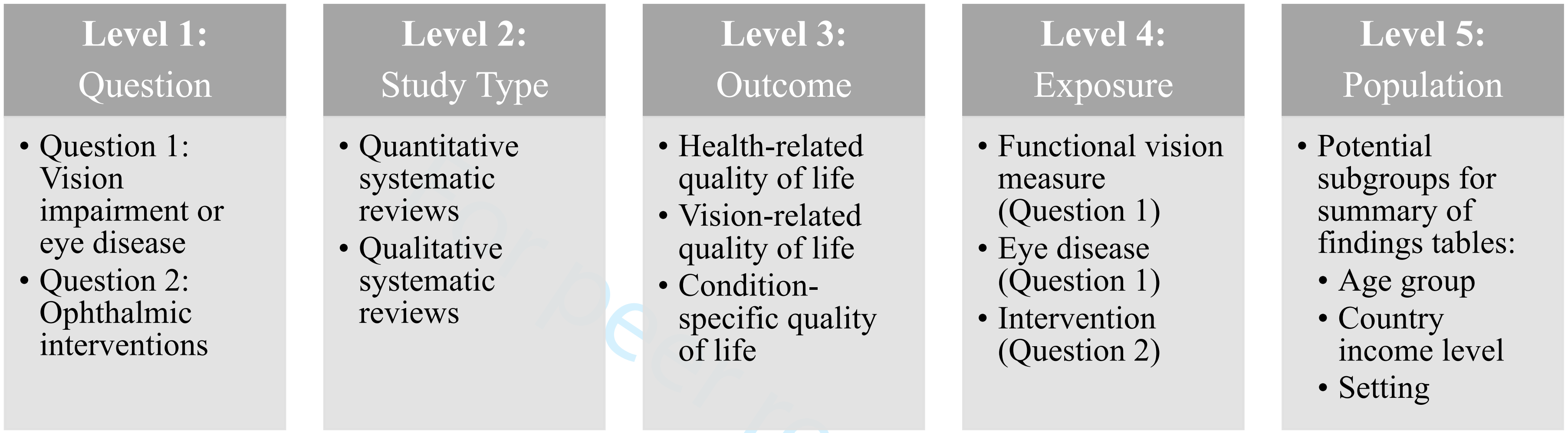

For peer review only

Online Supplementary File 1

Reporting checklist for protocol of a systematic review
Based on the PRISMA-P guidelines.

		Reporting Item	Page Number
**Title**			
Identification	#1a	Identify the report as a protocol of a systematic review	1
Update	#1b	If the protocol is for an update of a previous systematic review, identify as such	NA
**Registration**			
	#2	If registered, provide the name of the registry (such as PROSPERO) and registration number	6
**Authors**			
Contact	#3a	Provide name, institutional affiliation, e-mail address of all protocol authors; provide physical mailing address of corresponding author	1
Contribution	#3b	Describe contributions of protocol authors and identify the guarantor of the review	11-12
**Amendments**			
	#4	If the protocol represents an amendment of a previously completed or published protocol, identify as such and list changes; otherwise, state plan for documenting important protocol amendments	6
**Support**			
Sources	#5a	Indicate sources of financial or other support for the review	12
Sponsor	#5b	Provide name for the review funder and / or sponsor	12
Role of sponsor or funder	#5c	Describe roles of funder(s), sponsor(s), and / or institution(s), if any, in developing the protocol	12
**Introduction**			

For peer review only

Rationale	#6	Describe the rationale for the review in the context of what is already known	5
Objectives	#7	Provide an explicit statement of the question(s) the review will address with reference to participants, interventions, comparators, and outcomes (PICO)	5
**Methods**			
Eligibility criteria	#8	Specify the study characteristics (such as PICO, study design, setting, time frame) and report characteristics (such as years considered, language, publication status) to be used as criteria for eligibility for the review	6-7
Information sources	#9	Describe all intended information sources (such as electronic databases, contact with study authors, trial registers or other grey literature sources) with planned dates of coverage	7-8
Search strategy	#10	Present draft of search strategy to be used for at least one electronic database, including planned limits, such that it could be repeated	7
Study records - data management	#11a	Describe the mechanism(s) that will be used to manage records and data throughout the review	8
Study records - selection process	#11b	State the process that will be used for selecting studies (such as two independent reviewers) through each phase of the review (that is, screening, eligibility and inclusion in meta-analysis)	8-9
Study records - data collection process	#11c	Describe planned method of extracting data from reports (such as piloting forms, done independently, in duplicate), any processes for obtaining and confirming data from investigators	9-10
Data items	#12	List and define all variables for which data will be sought (such as PICO items, funding sources), any pre-planned data assumptions and simplifications	9-10
Outcomes and prioritization	#13	List and define all outcomes for which data will be sought, including prioritization of main and additional outcomes, with rationale	9-10

For peer review only

Risk of bias in individual studies	#14	Describe anticipated methods for assessing risk of bias of individual studies, including whether this will be done at the outcome or study level, or both; state how this information will be used in data synthesis	9-11
Data synthesis	#15a	Describe criteria under which study data will be quantitatively synthesised	NA
Data synthesis	#15b	If data are appropriate for quantitative synthesis, describe planned summary measures, methods of handling data and methods of combining data from studies, including any planned exploration of consistency (such as I2, Kendall's τ)	NA
Data synthesis	#15c	Describe any proposed additional analyses (such as sensitivity or subgroup analyses, meta-regression)	NA
Data synthesis	#15d	If quantitative synthesis is not appropriate, describe the type of summary planned	10-11
Meta-bias(es)	#16	Specify any planned assessment of meta-bias(es) (such as publication bias across studies, selective reporting within studies)	NA
Confidence in cumulative evidence	#17	Describe how the strength of the body of evidence will be assessed (such as GRADE)	11

The PRISMA-P checklist is distributed under the terms of the Creative Commons Attribution License CC-BY 4.0. This checklist was completed on 06. February 2020 using https://www.goodreports.org/, a tool made by the EQUATOR Network in collaboration with Penelope.ai

For peer review only

Online Supplementary File 2

Search strategy for MEDLINE Ovid

1. exp Eye Diseases/

2. exp Eye Injuries/

3. exp Administration, Ophthalmic/

4. exp Diagnostic Techniques, Ophthalmological/

5. exp Eye Protective Devices/

6. exp Glaucoma Drainage Implants/

7. exp Injections, Intraocular/

8. exp Ophthalmic Solutions/

9. exp Ophthalmologic Surgical Procedures/

10. exp Optical Devices/

11. exp Orbital Implants/

12. exp Orthoptics/

13. exp Pseudophakia/

14. exp Visual Prosthesis/

15. ((low* or handicap* or subnormal* or impair* or partial* or disab* or reduce* or diminish* or decrease*) adj3 (vision or visual* or sight*)).tw.

16. ((abnormal* or blurred or defect* or difficult* or dim or disturbed or hazy or interference or poor or tunnel or weak* or defect* or deficienc* or disorder* or disturb* or problem*) adj3 (vision or visual* or sight*)).tw.

17. ((delayed or agnosia or constriction* or prosthesis or prostheses) adj3 (vision or visual* or sight*)).tw.

18. ((vision or visual or sight*) adj2 loss).tw.

19. (Ocular or occular or intraocular or ophthalmol* or ophthalmic* or ophthalmop* or optic* or orbital or conjunctival or conjunctivitis or eye or eyes or eyelid* or cataract* or corneal or glaucoma* or lacrimal or lacrimation or macular or retinal or retinitis or retinoblastoma or retinopath* or retrobulbar neuritis or uveal or uveitis or vitrectomy or vitreous detachment* or vitreous haemorrhage* or vitreous hemorrhage* or vitreous membranes or vitreous strands or vitreous prolapse* or vitreous syneresis).tw.

For peer review only

20. (Amblyopia or Ametropia or Anisocoria or Anophthalmia or Anterior Chamber Haemorrhage or Anterior Chamber Hemorrhage or Aphakia or Aqueous Outflow Obstruction or Asthenopia or Balint's Syndrome or Blepharitis or Blepharospasm or chalazia or chalazion or Chorioretinal Disorder* or Chorioretinitis or Choroid Diseases or Choroidal or Choroiditis or Chromatopsia or Diplopia or Endophthalmitis or Epiphora or Episcleritis or Equatorial Staphyloma or Esotropia or Exophthalmos or Fixed Pupil* or Fuchs endothelial dystrophy or Hemianopia or Hemianopsia or Hepatolenticular Degeneration or Hordeola or Hordeolum or Horner's Syndrome or Hypopyon or Iritis or Keratitis or Keratoconjunctivitis or Keratoconus or Lens Disease* or Lens Disorder* or Lens Opacit* or Lens Subluxation or Localized Anterior Staphyloma or Meibomianitis or Miosis or Mydriasis or Myopia or Nystagmus or Oculopath* or Papilloedema or Periorbital Fat Herniation or (Periocular and carcinoma*) or Photalgia or Photophobia or Photopsia or Pigment Precipitation or Posterior capsule opacification or Posterior Dislocation Of Lens or Posterior Synechiae or Pseudophakia or Proliferative Vitreoretinopathy or Scleral Disease* or Scleral Staphyloma or Scleritis or Scotoma or Staphyloma Posticum or Strabismus or Symblepharon or Traumatic Hyphema or Wavefront Aberration* or Wegener's granulomatosis or Wilson's Disease or Xerophthalmia or refractive error* or near-sighted* or nearsighted* or short-sighted* or shortsighted* or hyperopia or farsighted* or far sighted* or long-sighted* or longsighted*or astigmatism or presbyopia* or onchocerciasis or onchocerciases).tw.

21. (LASIK or LASEK or Orthoptic* or "visual prosthesis" or "visual prostheses" or "artificial iris" or "capsular tension ring" or "cornea implant" or "intravitreal implant" or "lens implant" or "palpebral spring" or "punctal plug" or "retinal implant" or "sclerectomy implant" or glasses or spectacle* or "artificial lens" or "artificial implant lens" or pseudophakos or "orbit implant" or "ab interno gel implant" or "ab interno gel stent" or "anterior chamber drainage tube" or "aqueous drainage device" or "aqueous drainage implant" or "aqueous shunt" or glaukos or istent or keratoprosthesis).tw.

22. or/1-21

23. "Quality of Life"/ or Quality-Adjusted Life Years/ or "Value of Life"/ or Health Status/ or Sickness Impact Profile/ or Disability Evaluation/ or exp "Activities of Daily Living"/ or Cost-Benefit Analysis/ or "Surveys and Questionnaires"/ or Health surveys/ or exp psychometrics/

24. (quality adj2 life).tw.

25. ("disability adjusted life" or qaly* or qald* or qale* or qtime* or daly* or euroqol or "euro qol" or eq5d or "eq 5d" or hql or hqol or "h qol" or hrqol or "hr qol" or hye or hyes or health* year* equivalent* or hui or hui1 or hui2 or hui3 or "willingness to pay" or "standard gamble" or QOL or HRQL or HRQOL or wellbeing or "well being" or WHOQOL or "healthy days measures" or "EQ VAS" or "EQ 15D" or "36 Item Short Form Survey" or "SF 36" or "12 item Short Form Survey" or "SF 12" or "Visual Function Questionnaire" or "NEI VFQ" or "VFQ 25" or "IND VFQ 33" or "14 item Visual Functioning" or "VF 14" or "11 item Visual Functioning" or "VF 11" or "Impact of Vision Impairment" or IVI or "glaucoma utility index" or catquest or "Activities of Daily Vision Scale" or ADVS or "Cataract Symptom Scale" or "Daily Living Tasks Dependent Upon Vision" or DLTV or "Measure of Outcome in Ocular Disease" or "Refractive Status and Vision Profile" or "Vision Specific Sickness Impact Profile" or SIPV or "Visual Activities Questionnaire*" or VAQ or "Visual

For peer review only

Disability Assessment*" or VDA or "Visual Disabilities Questionnaire*" or "Visual Function Questionnaire*" OR "VA LV VFQ" or "Glaucoma symptom scale" or "Symptom Impact Glaucoma Score" or GHPI or "Glaucoma Health Perceptions index").tw.

26. (health adj3 (utility* or disutili* or state or status)).tw.

27. ((visual or vision) adj3 (disabilit* or disabled or function* or activit* or task or performance or impairment or Questionnaire*)).tw.

28. or/23-27

29. 22 and 28

30. Cochrane Database Syst Rev.ja. or Meta-Analysis.pt. or (Search* or Medline or (Systematic and Review)).tw.

31. limit 29 to systematic reviews

32. 30 or 31

33. exp Animals/ not exp Humans/

34. 32 not 33

35. 29 and 34

For peer review only

Online Supplementary File 3

Joanna Briggs Institute Data Extraction Form for Review for Systematic Reviews and Research Syntheses

Study Details	
Author/Year	
Objectives	
Participants	
Characteristics	
Total number	
Setting/context	
Description of Interventions/phenomena of interest	
Search Details	
Sources searched	
Range (years) of included studies	
Number of studies included	
Types of studies included	
Country of origin of included studies	
Appraisal	
Appraisal instruments used	
Appraisal rating	
Analysis	
Method of analysis	
Outcome assessed	
Results/Findings	
Significance/direction	
Heterogeneity	
Comments	

For peer review only
